# The HopQ-CEACAM Interaction Controls CagA Translocation, Phosphorylation, and Phagocytosis of *Helicobacter pylori* in Neutrophils

**DOI:** 10.1128/mBio.03256-19

**Published:** 2020-02-04

**Authors:** Ina-Kristin Behrens, Benjamin Busch, Hellen Ishikawa-Ankerhold, Pia Palamides, John E. Shively, Cliff Stanners, Carlos Chan, Nelly Leung, Scott Gray-Owen, Rainer Haas

**Affiliations:** a Chair of Medical Microbiology and Hospital Epidemiology, Max von Pettenkofer Institute, Faculty of Medicine, LMU Munich, Munich, Germany; b Department of Internal Medicine I, Faculty of Medicine, LMU Munich, Munich, Germany; c Department of Molecular Imaging and Therapy, Beckman Research Institute, City of Hope, Duarte, California, USA; d Department of Biochemistry and Goodman Cancer Research Centre, McGill University, Montreal, Quebec, Canada; e Division of Surgical Oncology and Endocrine Surgery, University of Iowa Carver College of Medicine, Iowa City, Iowa, USA; f Department of Molecular Genetics, University of Toronto, Toronto, Ontario, Canada; g German Center for Infection Research (DZIF), LMU Munich, Germany; New York University School of Medicine

**Keywords:** *Helicobacter pylori*, *cag*-type IV secretion system, CagA translocation, tyrosine-phosphorylation, phagocytosis, neutrophils, PMNs, humanized CEACAM myeloid cells, CEACAM, CEACAM-humanized mice, CagA, *Helicobacter*

## Abstract

The *cag* type IV secretion system (*cag*-T4SS) of Helicobacter pylori exploits specific cellular carcinoembryonic antigen-related cell adhesion molecules (CEACAMs), such as CEACAM1, -3, -5, and -6, as cellular receptors for CagA translocation into human gastric epithelial cells. We studied the interaction of H. pylori with human CEACAM1, CEACAM3, and CEACAM6 receptors (hCEACAMs) expressed on myeloid cells from CEACAM-humanized mice. Human and CEACAM-humanized mouse polymorphonuclear neutrophils (PMNs) allowed a specific HopQ-dependent interaction strongly enhancing CagA translocation. Translocated CagA was tyrosine phosphorylated, which was not seen in wild-type (wt) murine neutrophils. In contrast, human or murine bone marrow-derived macrophages and dendritic cells (DCs) revealed a low hCEACAM expression and bacterial binding. CagA translocation and tyrosine-phosphorylation was low and independent of the HopQ-CEACAM interaction. Neutrophils, but not macrophages or DCs, from CEACAM-humanized mice, significantly upregulated the proinflammatory chemokine MIP-1α. However, macrophages showed a significantly reduced amount of CXCL1 (KC) and CCL2 (MCP-1) secretion in CEACAM-humanized versus wt cells. Thus, H. pylori, via the HopQ-CEACAM interaction, controls the production and secretion of chemokines differently in PMNs, macrophages, and DCs. We further show that upon H. pylori contact the oxidative burst of neutrophils and phagocytosis of H. pylori was strongly enhanced, but hCEACAM3/6 expression on neutrophils allowed the extended survival of H. pylori within neutrophils in a HopQ-dependent manner. Finally, we demonstrate that during a chronic mouse infection, H. pylori is able to systemically downregulate hCEACAM1 and hCEACAM6 receptor expression on neutrophils, probably to limit CagA translocation efficiency and most likely gastric pathology.

## Abstract (precis)

Helicobacter pylori is highly adapted to humans and evades host immunity to allow its lifelong colonization. However, the H. pylori mouse model is artificial for H. pylori, and few adapted strains allow gastric colonization. Here, we show that human or CEACAM-humanized, but not mouse neutrophils are manipulated by the H. pylori HopQ-CEACAM interaction. Human CEACAMs are responsible for CagA phosphorylation, activation, and processing in neutrophils, whereas CagA translocation and tyrosine phosphorylation in DCs and macrophages is independent of the HopQ-CEACAM interaction. H. pylori affects the secretion of distinct chemokines in CEACAM-humanized neutrophils and macrophages. Most importantly, human CEACAMs on neutrophils enhance binding, oxidative burst, and phagocytosis of H. pylori and enhance bacterial survival in the phagosome. The H. pylori-CEACAM interaction modulates PMNs to reduce the H. pylori CagA translocation efficiency *in vivo* and to fine-tune the expression of CEACAM receptors on neutrophils to limit translocation of CagA and gastric pathology.

## INTRODUCTION

Gastric infection with Helicobacter pylori is still a major cause for chronic gastritis and gastroduodenal ulcers, but it is also a risk factor for the development of mucosa-associated lymphoid tissue lymphoma and gastric adenocarcinoma (1, 2). Two major bacterial virulence factors are especially associated with gastric disease induction by H. pylori, the vacuolating toxin VacA (3) and a 40-kb pathogenicity island (*cag*-PAI) (4). The *cag*-PAI harbors a type IV secretion system (T4SS), which is used by H. pylori to inject the 120- to 145-kDa immunodominant cytotoxin-associated gene A (CagA), as well as the lipopolysaccharide (LPS) metabolite heptose-1,7-bisphosphate (5–7) into different types of host cells (8, 9). The presence of a functional *cag*-PAI is associated with the development of severe gastric inflammation, the occurrence of gastric or duodenal ulcers, and gastric cancer (1).

The structure and function of the *cag*-T4SS is still not well understood, but recent electron microscopy data identified a T4SS core complex (10). These extracellular appendages were also described earlier as T4SS pili (11–13). The T4SS-encoded proteins CagI, CagL, CagY, and CagA are known to interact with β1 integrin receptors on the cell surface, which were originally suggested to support the delivery of the CagA toxin into target cells (12, 13). However, recent evidence, based on a CRISPR/Cas-mediated integrin gene deletion strategy, revealed that the H. pylori*-*integrin interaction is not necessary for CagA translocation (14). Notably, besides the β1 integrin interaction, H. pylori also targets various receptors of the human carcinoembryonic antigen-related cell adhesion molecule (CEACAM) family (CEACAM1, CEACAM3, CEACAM5, and CEACAM6) (15, 16). CEACAMs represent a subset of the immunoglobulin (Ig) superfamily of proteins and consist of a variable number of Ig-like constant domains and an Ig variable domain-like N terminus that allows binding to adhesins of different bacterial origin.

H. pylori is highly adapted to colonization of the human stomach mucosa. The unique H. pylori outer membrane adhesin HopQ (HP1177), which is not part of the *cag*-PAI, mediates the specific bacterial adhesion to CEACAMs, which is essential for a successful delivery of CagA into epithelial target cells (15–17). Recent structural studies have shown that HopQ disrupts transdimerization in human CEACAMs and uses a coupled folding and binding mechanism to engage the canonical CEACAM dimerization interface for CEACAM recognition and CagA translocation. A cellular signaling event via the cytoplasmic tail of CEACAMs was not necessary for CagA translocation into epithelial cells (18, 19).

A number of other human-specific Gram-negative bacteria, such as Neisseria gonorrhoeae, Haemophilus influenzae, Moraxella catarrhalis, and uropathogenic Escherichia coli specifically adhere to human CEACAMs. CEACAM1 is most widely expressed and found on epithelial cells but also on certain endothelial, lymphocytic, and myeloid cells, whereas CEACAM3 is restricted to neutrophils, and CEACAM6 is found on epithelial and myeloid cells. CEACAM1 contains a cytoplasmic immunoreceptor tyrosine-based inhibitory motif, which obviously can be exploited by certain bacteria to suppress T cell (20), B cell (21), dendritic cell (DC) (22), and epithelial cell (23) responses (reviewed in reference 24). CEACAM3, which is human-restricted and contains an immunoreceptor tyrosine-based activation motif (ITAM), is expressed on neutrophils and lacks a cell adhesion function but can trigger an efficient opsonin-independent phagocytosis of bacteria (25, 26). Thus far, the H. pylori-CEACAM interaction has only been studied for epithelial cells, but virtually nothing is known about the importance and the consequences of a HopQ-CEACAM interaction with cells of the host immune system.

H. pylori does not interact with CEACAMs from other mammals, including mice, although certain H. pylori isolates could be successfully adapted to colonize the gastric mucosa of mice (mouse-adapted strains, such as the Sydney strains SS1 and PMSS1). However, mouse infections do not recapitulate the strong gastric inflammation and pathology eventually seen in humans. Thus, current mouse-adapted H. pylori strains either do not possess a functional *cag*-T4SS (SS1) (27) or tend to rapidly switch it off (PMSS1) (28, 29).

Here, we studied the effect of H. pylori on immune cells, especially neutrophils, macrophages, and DCs *in vitro*. We compared conventional murine cells with the corresponding human counterparts and murine myeloid cells expressing human CEACAMs (CEACAM-humanized immune cells). We analyzed their interaction with H. pylori, the translocation and phosphorylation of CagA, and the potential consequences of this interaction with regard to chemokine release and phagocytosis.

## RESULTS

### Breeding of mouse lines, isolation of CEACAM-humanized mouse myeloid cells, and their characterization for CEACAM expression levels.

The inflammatory response induced by H. pylori infection of humans, but not of mice, is characterized by a large influx of inflammatory cells to the lamina propria of the gastric mucosa (30, 31), and neutrophils are usually the first cells to arrive at the site of infection. For studies of the interaction of H. pylori with human, mouse, and mouse CEACAM-humanized immune cells, we isolated and purified bone marrow-derived polymorphonuclear neutrophils (PMNs), murine bone marrow-derived macrophages, and bone marrow-derived DCs from wild-type (wt) and humanized CEACAM1-transgenic C57BL/6 mice (Tg418, expression of human CEACAM1 in myeloid cells, here termed hCEACAM1). Furthermore, CEACAM3/5/6/7 transgenic mice (CEABAC10, expression of hCEACAM3 and -6 in myeloid cells) and the combination of both generated by cross-breeding (hCEACAM1, -3, and -6 in myeloid cells; here termed hCEACAM_all_) were used for cell isolation.

Next, all mouse lines were crossed with the C57BL/6 2D2 mouse line (32) carrying an inactivation of the murine CEACAM1 gene to eliminate an interference between the original murine CEACAM1 and the transgenic version. To exclude negative effects concerning the surface expression of the human CEACAM receptors due to the intensive cross breeding of the mouse lines, we first analyzed the level of hCEACAM expression by the different transgenic myeloid cells by flow cytometry using human-specific anti-CEACAM antibodies (see Table S1 in the supplemental material for antibodies).

10.1128/mBio.03256-19.8 TABLE S1 Antibodies used in this study and their sources. Download Table S1, DOCX file, 0.01 MB. Copyright © 2020 Behrens et al. 2020 Behrens et al. This content is distributed under the terms of the Creative Commons Attribution 4.0 International license.

**FIG 1:**
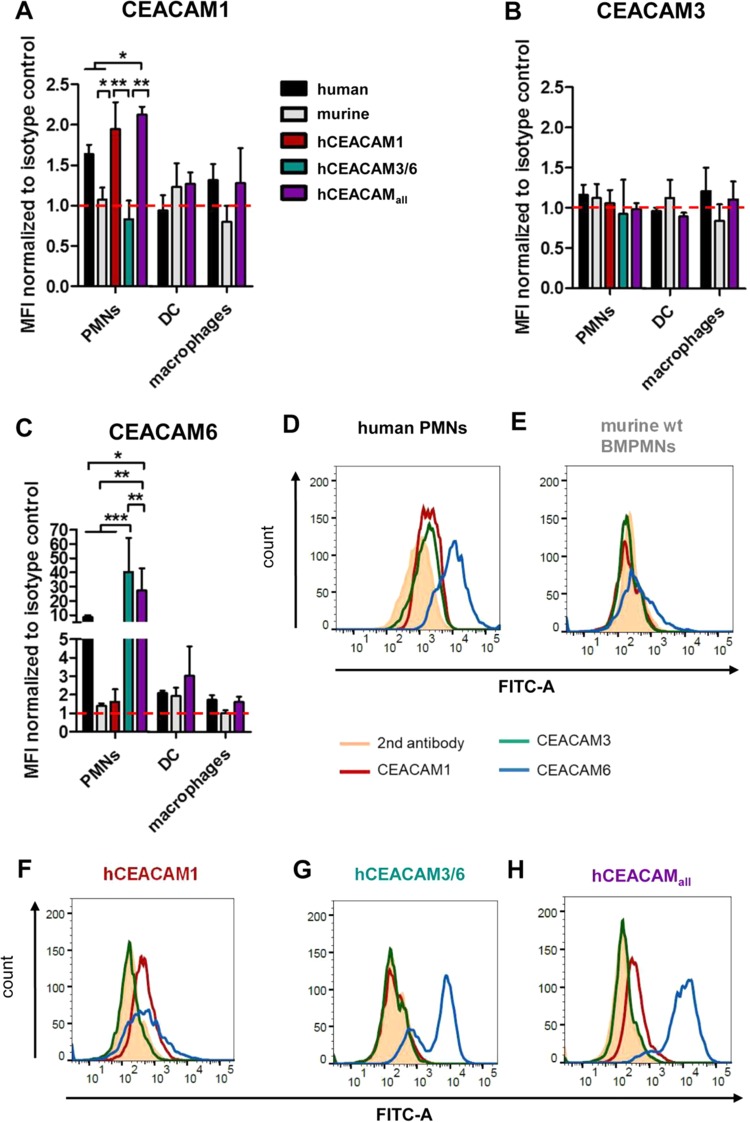
Expression of human CEACAMs on the cell surfaces of isolated human, murine, and CEACAM-humanized myeloid cells. (A to C) Determination of hCEACAM1, hCEACAM3, or hCEACAM6 production by PMNs, DCs, and macrophages of human or mouse origin as determined by flow cytometry. Mean fluorescence intensity (MFI) was normalized to isotype control. (D to H) PMNs were obtained from human blood (D) or isolated from wt mouse bone marrow (E) or from various CEACAM-humanized mouse bone marrow preparations (F to H). The cells were fixed and stained with antibodies specific for human CEACAM1, hCEACAM3, hCEACAM6, or a mouse IgG isotype control and analyzed by flow cytometry. Isotype histograms are shaded. Representative plots of experiments performed three times are shown. Data were analyzed by two-way ANOVA and the Bonferroni *post hoc* test. *, *P* < 0.05; **, *P* < 0.01; ***, *P* < 0.001.

A low level of CEACAM1 production was generally seen on the cell surface of both human and CEACAM1-humanized murine neutrophils, but no hCEACAM was detected in wt mouse neutrophils (Fig. 1A). For murine DCs no hCEACAM1 expression was measurable, and humanized mouse DCs (hCEACAM_all_) also did not express significant levels of human CEACAM1 (Fig. 1A). Isolated human macrophages produced low levels of hCEACAM1, similar to CEACAM1-humanized mouse macrophages, but no hCEACAM1 expression was detectable in wt murine macrophages (Fig. 1A). hCEACAM3 expression was generally very low or even nondetectable in all mouse wt and CEACAM-humanized cells tested (Fig. 1B), which is in agreement with results described earlier (33). The most abundant surface localization was seen for human CEACAM6 in human neutrophils and even higher in CEACAM-humanized mouse neutrophils (Fig. 1C, D, G, and H), whereas human, mouse, and CEACAM-humanized DCs and macrophages did not produce significant levels of CEACAM6 (Fig. 1C). The identity of isolated and differentiated murine PMNs, DCs, and macrophages was verified by flow cytometry analysis, demonstrating the expression of Ly6G in combination with CD11b (PMNs), CD11c (DCs) or F4/80 (macrophages) for the corresponding myeloid cell types (Fig. S1A to D), or stained murine PMNs with Giemsa (Fig. S1E). In conclusion, these results suggest that murine neutrophils surface express hCEACAM1 modestly and hCEACAM 6 robustly, but not hCEACAM3. In contrast, murine DCs and macrophages did not express any of the hCEACAMs.

10.1128/mBio.03256-19.1 FIG S1 Detection of characteristic markers for PMNs, DCs, and macrophages and expression of human CEACAMs on mouse transgenic neutrophils, DCs, and macrophages. (A to D) To verify the quality of isolated PMNs, DCs, and macrophages from human and mouse origins, the expression of defined characteristic markers on these cells was tested by flow cytometry using the following antibodies: APC rat anti-mouse Ly6G clone 1A8 (BioLegend); FITC anti mouse/human CD11b clone M1/70 (BioLegend); APC anti human CD66abce clone TET2 (Miltenyi Biotec); rat IgG2a kappa isotype control, APC clone eBR2a (BioLegend); Rat IgG2a kappa isotype control FITC (BD Bioscience). (D) Giemsa staining of murine neutrophils showing the typical staining pattern with segmentation. Download FIG S1, PDF file, 0.4 MB. Copyright © 2020 Behrens et al. 2020 Behrens et al. This content is distributed under the terms of the Creative Commons Attribution 4.0 International license.

### Human and CEACAM-humanized mouse neutrophils, but not murine macrophages or DCs, show a specific HopQ-dependent interaction with *H. pylori.*

**FIG 2:**
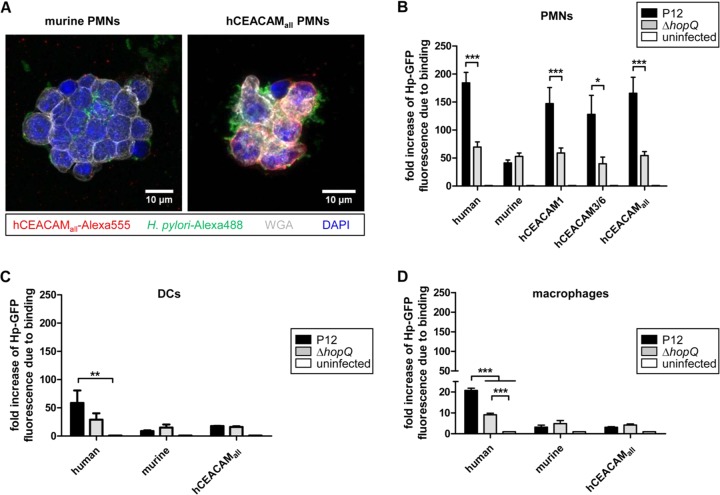
Human and CEACAM-humanized mouse neutrophils, rather than macrophages or DCs, bind H. pylori in a HopQ-dependent manner. (A) Micrographs showing the specific binding of H. pylori to murine wt or CEACAM-humanized PMNs expressing human CEACAM receptors. The micrograph shows a merge of CEACAM_all_ (red) H. pylori (green) membranes stained by wheat germ agglutinin (WGA; gray) and nuclei with DAPI (blue). For single stainings, see Fig. S6. Binding of human, murine, or CEACAM-humanized PMNs (B), DCs (C), and macrophages (D) to H. pylori P12-GFP or P12Δ*hopQ*-GFP was determined by flow cytometry. For all assays, infection was for 1 h at an MOI of 10. *n* ≥ 3 for all experiments. The data were normalized to uninfected cells and GFP expression. Data were analyzed by two-way ANOVA and the Bonferroni *post hoc* test. *, *P* < 0.05; **, *P* < 0.01; ***, *P* < 0.001.

We next were interested whether the different CEACAM-humanized neutrophils, macrophages, and DCs would show an altered behavior in their interaction with H. pylori compared to the corresponding wt human or murine myeloid cells. CEACAM-humanized (hCEACAM_all_) but not murine neutrophils showed a strong binding and engulfment of the H. pylori P12-GFP strain by fluorescence microscopy (Fig. 2A). Quantification of the binding and engulfment events by a flow cytometry approach supported the microscopic data and revealed a generally lower interaction of murine PMNs with H. pylori, independent of the HopQ adhesin (Fig. 2B). However, both human and the different hCEACAM-expressing murine neutrophils exhibited a stronger binding to H. pylori compared to murine wt PMNs (Fig. 2B). A similar stronger binding of CEACAM-humanized mouse neutrophils compared to wt neutrophils was also measured with a set of other, independent H. pylori strains (Fig. S2), suggesting a general low binding capacity of H. pylori to murine wt PMNs. Taken together, these results suggest a stronger recognition of H. pylori by human or CEACAM-humanized neutrophils compared to wt murine neutrophils.

10.1128/mBio.03256-19.2 FIG S2 Independent H. pylori strains binding to CEACAM-humanized mouse neutrophils. Mouse wt and CEACAM-humanized mouse neutrophils were used for binding of different H. pylori wt, as well as Δ*hopQ* mutant strains. Binding of PMNs (murine or CEACAM-humanized) to H. pylori P12-GFP or P12Δ*hopQ*-GFP (MOI of 10; 1 h) was determined by flow cytometry. (*n* = 3). Statistics: two-way ANOVA and the Bonferroni *post hoc* test. *, *P* < 0.05; **, *P* < 0.01; ***, *P* < 0.001. Download FIG S2, PDF file, 0.3 MB. Copyright © 2020 Behrens et al. 2020 Behrens et al. This content is distributed under the terms of the Creative Commons Attribution 4.0 International license.

The interaction of H. pylori P12-GFP with DCs or macrophages was generally much lower compared to neutrophils, possibly reflecting the lower expression of hCEACAMs in DCs and macrophages compared to neutrophils. Notably, there was no significant difference between P12-GFP and P12Δ*hopQ*-GFP in binding to DCs but to human macrophages (Fig. 2C and D), suggesting that hCEACAM6, which was most strongly expressed by human and CEACAM-humanized PMNs (Fig. 1C), was not sufficiently expressed on DCs (Fig. 1C). In conclusion, our *in vitro* interaction data show that from the set of myeloid cells tested H. pylori interacted preferentially with human or CEACAM-humanized mouse neutrophils in a HopQ-dependent way.

### CEACAMs are responsible for CagA phosphorylation, activation, and processing in neutrophils.

**FIG 3:**
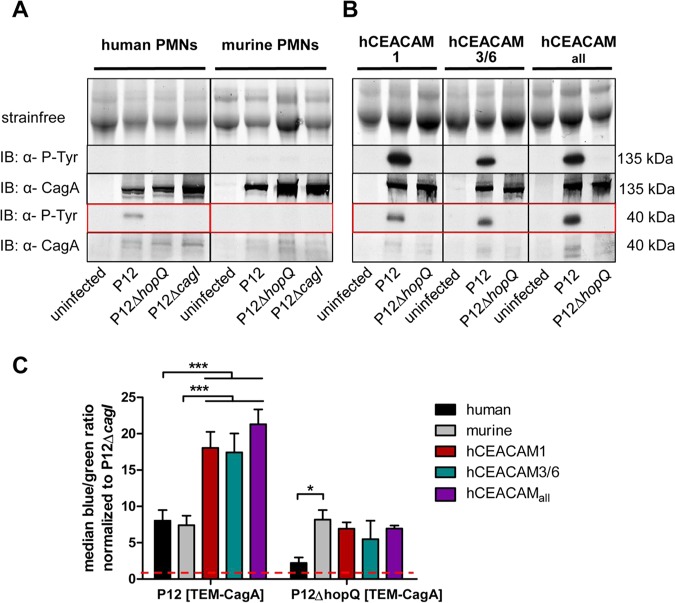
Translocation of CagA into murine bone marrow-derived PMNs and CagA tyrosine-phosphorylation is specifically enhanced by the HopQ-CEACAM interaction of CEACAM-humanized neutrophils. (A) Human or murine PMNs were infected with strain P12, strain P12Δ*hopQ*, or the P12Δ*cagI* for 2.5 h with an MOI of 60. Translocation of CagA was determined by detecting tyrosine-phosphorylated CagA (α-P-Tyr) with antibody PY99 in the immunoblot. (B) Human, mouse, or CEACAM-humanized murine PMNs (hCEACAM1, hCEACAM3/6, and hCEACAM_all_) were infected with TEM H. pylori strain P12, strain P12Δ*hopQ*, or the P12Δ*cagI* strain (3 h; MOI of 60). Translocation of CagA was determined by detecting tyrosine-phosphorylated CagA (α-P-Tyr) with the antibody PY99 in the immunoblot. Phosphorylated CagA is (partially) processed in PMNs and appears as a full-length form (∼135 kDa) or as a C-terminal fragment of ∼40 kDa (see red box), as shown for all blots. The Stain-Free method was used as loading control for all immunoblotting experiments (49). (C) Quantitative evaluation of CagA translocation into human, murine, and CEACAM-humanized PMNs by the TEM assay. PMNs were infected with H. pylori P12[TEM-CagA] or P12Δ*hopQ*[TEM-CagA] and normalized against the translocation-deficient P12Δ*cagI*[TEM-CagA] deletion mutant. The red line marks the level of the normalized P12Δ*cagI* controls (*n* = 4). Data were assessed using two-way ANOVA and the Bonferroni *post hoc* test. *, *P* < 0.05; **, *P* < 0.01; ***, *P* < 0.001.

We next investigated the ability of H. pylori for translocation of its effector protein CagA into murine neutrophils and its activation by tyrosine phosphorylation. CagA was injected into human neutrophils and was cleaved into an ∼40-kDa tyrosine-phosphorylated product (Fig. 3A). The full-length tyrosine-phosphorylated CagA (∼135 kDa) was not detectable, suggesting its specific, rapid, and complete processing in human neutrophils, as described earlier (9, 34). Surprisingly, in murine neutrophils CagA was not detectable as a phosphorylated protein (Fig. 3A), raising the question of whether translocation of CagA into mouse PMNs did not occur or whether its tyrosine phosphorylation and specific detection did not work. An *in vitro* phosphorylation assay, in which lysed mouse PMNs were coincubated with H. pylori and analyzed by immunodetection, revealed full-length tyrosine-phosphorylated CagA, suggesting that the functional kinases were available and active in these cells (Fig. S3). In contrast to wt mouse PMNs, all CEACAM-humanized PMNs carrying different combinations of hCEACAM receptors were fully competent for CagA translocation and tyrosine phosphorylation (Fig. 3B). The immunoblots show a full-length CagA (∼135 kDa), as well as the processed C-terminal CagA fragment (∼40 kDa), in CEACAM-humanized PMNs in high quantities.

10.1128/mBio.03256-19.3 FIG S3 *In vitro* CagA phosphorylation assay. H. pylori wt and mutant strains were used for infection experiments of murine PMNs (MOI of 60; 10 min). Cell lysates of infected PMNs and bacterial lysates were used for immunoblotting using antibodies as indicated. Download FIG S3, PDF file, 0.2 MB. Copyright © 2020 Behrens et al. 2020 Behrens et al. This content is distributed under the terms of the Creative Commons Attribution 4.0 International license.

We next applied the quantitative β-lactamase-dependent CagA reporter assay (TEM assay) to corroborate the immunoblotting results. The TEM assay determines H. pylori CagA translocation into host cells directly, rather than CagA tyrosine phosphorylation by detecting the cleavage a fluorescent dye due to the enzymatic activity of a translocated CagA-β-lactamase fusion protein (35). Notably, this assay clearly demonstrated a HopQ-independent CagA translocation into wt mouse PMNs at a similar level as seen for human PMNs (Fig. 3C), suggesting that CagA translocated in the absence of hCEACAMs is precluded from tyrosine-phosphorylation, or is rapidly dephosphorylated. CEACAM-humanized PMNs revealed a significantly elevated (2- to 3-fold) CagA translocation rate (Fig. 3C), but also a much stronger tyrosine phosphorylation, compared to human PMNs (compare Fig. 3A versus Fig. 3B), illustrating a physiological role for the human receptors.

### DCs and macrophages allow CagA translocation and tyrosine phosphorylation independent of the HopQ-CEACAM interaction.

**FIG 4:**
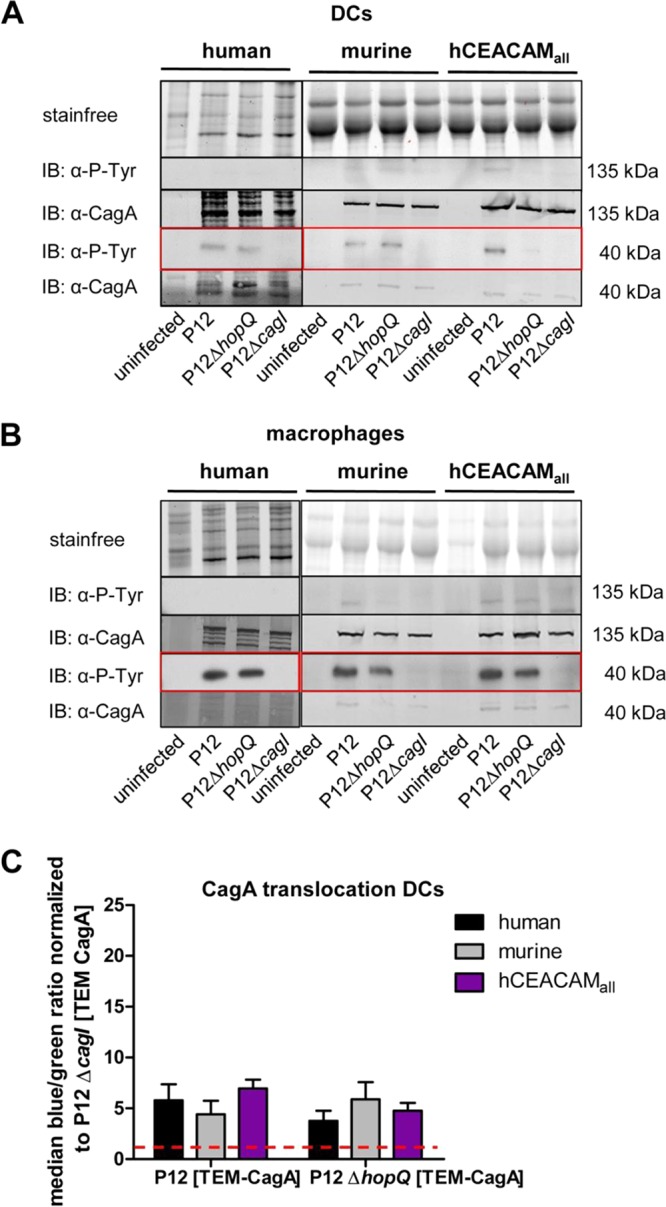
CagA translocation and its activation by tyrosine phosphorylation in human, mouse, and CEACAM-humanized DCs and macrophages is independent of the HopQ-CEACAM interaction. Monocytes isolated from human blood or bone marrow of wt and CEACAM-humanized mice were differentiated to DCs (A) and macrophages (B) and infected with strain P12, strain P12Δ*hopQ*, or the P12Δ*cagI* strain for 3 h at an MOI of 60. Translocation of CagA was determined by detecting tyrosine-phosphorylated CagA (α-P-Tyr) with antibody PY99 or CagA with antibody AK299 in the immunoblot. The Stain-Free method was used as a loading control for immunoblotting experiments (49). Full-length tyrosine-phosphorylated or nonphosphorylated forms of CagA (∼135 kDa) or the processed C-terminal fragment (∼40 kDa) (see red box) are shown for all blots. (C) Quantitative evaluation of CagA translocation into human, murine, and CEACAM-humanized DCs by the TEM assay. DCs were infected with H. pylori P12[TEM-CagA] or the P12Δ*hopQ*[TEM-CagA] strain and normalized against the translocation-deficient P12Δ*cagI*[TEM-CagA] deletion mutant at an MOI of 60 (red dotted line) (*n* ≥ 3). Data were assessed using two-way ANOVA and the Bonferroni *post hoc* test. *, *P* < 0.05; **, *P* < 0.01; ***, *P* < 0.001.

We next analyzed the ability of H. pylori to inject CagA into human and mouse DCs and macrophages. Tyrosine phosphorylation-specific immunoblots revealed that translocation and tyrosine phosphorylation of CagA was obtained for both DCs and macrophages of human, murine, and CEACAM-humanized origin (Fig. 4A and B). Phosphorylated CagA was processed as seen for neutrophils. Expectedly, tyrosine phosphorylation of CagA was independent of the HopQ interaction but dependent on the *cag*-T4SS in these myeloid cells (Fig. 4A and B).

Quantitative data generated by the TEM assay proved a low but comparable level of injected CagA for human and mouse DCs (Fig. 4C), which corroborated the results from the immunoblotting data (Fig. 4A). In accordance with the immunoblotting results, the quantification of CagA translocation into human and mouse DCs revealed similar CagA translocation rates for H. pylori P12 and the P12Δ*hopQ* mutant strain, suggesting that this translocation and phosphorylation of CagA was independent of the HopQ-CEACAM interaction. In contrast to DCs, murine macrophages were not suitable for the TEM assay (Fig. S4).

10.1128/mBio.03256-19.4 FIG S4 Comparison of the TEM-CagA assay for murine DCs and macrophages. Murine DCs and murine macrophages were infected with P12[TEM-CagA] (MOI of 60; 2.5 h), cells were loaded with CCF4-AM, fixed, and analyzed by fluorescence and brightfield microscopy. Download FIG S4, PDF file, 0.1 MB. Copyright © 2020 Behrens et al. 2020 Behrens et al. This content is distributed under the terms of the Creative Commons Attribution 4.0 International license.

Taken together, these data clearly demonstrate that, in contrast to PMNs, CagA translocation and tyrosine phosphorylation into mouse and human DCs and macrophages is largely independent from the HopQ-CEACAM interaction.

### *H. pylori* HopQ affects the secretion of distinct chemokines in CEACAM-humanized neutrophils and macrophages but not in DCs.

To determine whether the expression of human CEACAMs on the cell surface of mouse immune cells would change the physiological behavior of these cells after contact with H. pylori, we performed chemokine measurements with a bead-based sandwich immunoassay (mouse proinflammatory chemokine panel; BioLegend, San Diego, CA). We analyzed PMNs, DCs, and macrophages from wt and hCEACAM_all_ mice, which were infected with H. pylori P12 or left uninfected. Escherichia coli lipopolysaccharide (LPS) was used as a positive control. The overall response pattern differed between the independent myeloid cell types, and an overview of the chemokine response pattern of these cells with and without H. pylori infection is shown in Fig. S5 in the supplemental material.

10.1128/mBio.03256-19.5 FIG S5 General pattern of chemokine expression of CEACAM-humanized PMNs, DCs or macrophages infected by H. pylori as determined by the LEGENDplex mouse proinflammatory chemokine 13-plex panel (BioLegend). Only 10 of 13 chemokines are shown; the other ones did not show a secretion signal. (A) Infection of murine (*n* = 2) or CEACAM-humanized (*n* = 4) PMNs with H. pylori P12 or LPS as positive control and determination of chemokine response. (B) Infection of murine or CEACAM-humanized DCs with H. pylori P12 and determination of chemokine response (*n* = 3). (C) Infection of murine or CEACAM-humanized macrophages with H. pylori P12 and determination of chemokine response (*n* = 3). Statistics: two-way ANOVA and the Bonferroni *post hoc* test. *, *P* < 0.05; **, *P* < 0.01; ***, *P* < 0.001. Download FIG S5, PDF file, 0.5 MB. Copyright © 2020 Behrens et al. 2020 Behrens et al. This content is distributed under the terms of the Creative Commons Attribution 4.0 International license.

**FIG 5:**
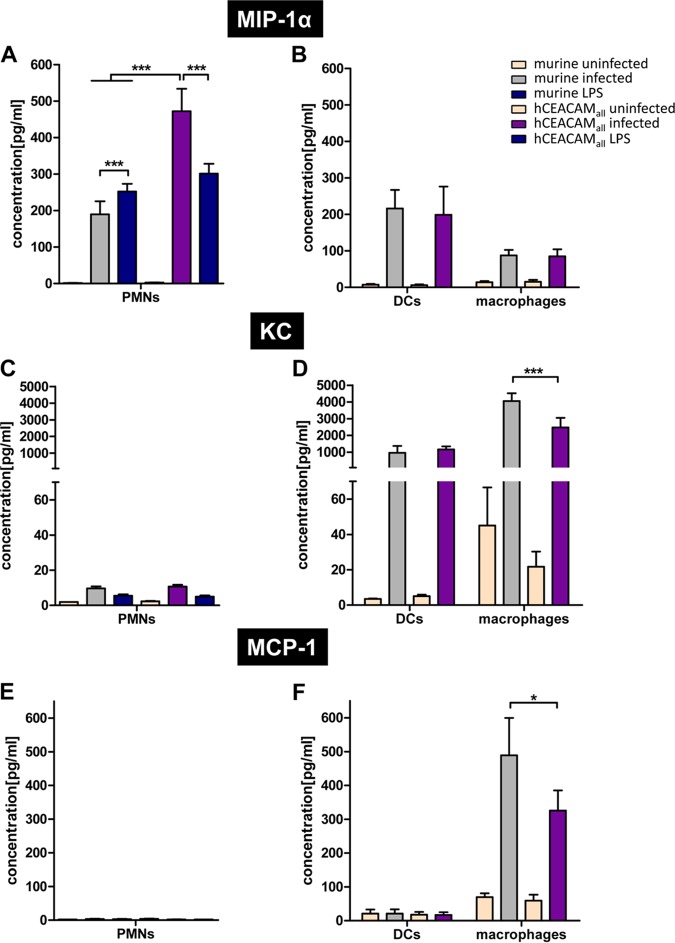
Effect of H. pylori-mediated binding to CEACAM-humanized PMNs, DCs, or macrophages on the secretion of the chemokines MIP-1α (CCL3), KC (CXCL1), and MCP-1 (CCL2). (A to C) Analysis of the production and secretion of MIP-1α (A), KC (B), or MCP-1 (C) from PMNs, DCs, or macrophages of wt or CEACAM-humanized mice left uninfected or infected for 3 h (MOI of 60) with H. pylori P12. Incubation of cells with 200 ng/ml LPS for 3 h was used as a positive control. Cells were analyzed with the LEGENDplex mouse proinflammatory chemokine 13-plex panel (BioLegend). Data were assessed using two-way ANOVA and the Bonferroni *post hoc* test. *, *P* < 0.05; **, *P* < 0.01; ***, *P* < 0.001.

H. pylori infection of mouse neutrophils induced a moderate secretion of the CC chemokine CCL3 (MIP-1α), which is generally involved in the recruitment and activation of PMNs through binding to the receptors CCR1, CCR4, and CCR5 (36). H. pylori infection of wt mouse PMNs induced a significantly lower signal than that obtained by treatment of the cells with E. coli LPS (positive control) (Fig. 5A). CEACAM-humanized PMNs infected by H. pylori significantly enhanced MIP-1α production, compared to wt murine PMNs or LPS-stimulated PMNs. This indicated that the murine cells expressing hCEACAMs *in vitro* have a distinct response to H. pylori infection, resulting in a local production of MIP-1α (Fig. 5A).

Interestingly, H. pylori induces a similar level of MIP-1α secretion in DCs compared to wt PMNs and a lower secretion in murine macrophages, whereas the secretion level of wt and CEACAM-humanized cells did not differ significantly in infected DCs and macrophages (Fig. 5A).

The chemokine CXCL1 (keratinocyte chemoattractant [KC]) is usually produced and secreted by macrophages, neutrophils, and epithelial cells and has neutrophil chemoattractant activity. KC was weakly upregulated upon H. pylori infection in PMNs but very strongly in DCs and macrophages (Fig. 5C and D). Whereas KC levels did not differ between wt and CEACAM-humanized DCs, a significant downregulation was observed in CEACAM-humanized compared to wt mouse macrophages (Fig. 5D). A similar situation was found for CCL2 (monocyte chemoattractant protein 1 [MCP-1]), which is supposed to recruit monocytes into foci of active inflammation and to regulate migration and tissue infiltration of macrophages (37). A significant downregulation of MCP-1 secretion was observed for H. pylori*-*infected CEACAM-humanized macrophages compared to wt mouse macrophages (Fig. 5F).

In conclusion, these data show that murine cells expressing hCEACAMs distinctly respond to H. pylori infection and can have very different effects on the regulation and/or secretion of important chemokines in different myeloid cell types. Our data suggest that a CEACAM interaction and eventually downstream signaling is most important in neutrophils, which also more strongly express CEACAMs on their surface, compared to DCs or macrophages (Fig. 1). MIP-1α was the only chemokine we found to be upregulated by the CEACAM-mediated signaling pathway. These data for murine macrophages and DCs are also supported by our CEACAM-independent H. pylori binding results (Fig. 2) and the CEACAM-independent CagA translocation and phosphorylation data (Fig. 4). DCs, which do not express any human CEACAMs, do not respond to H. pylori binding via HopQ, whereas human and CEACAM-humanized macrophages, showing a weak expression of hCEACAM1, do respond moderately to H. pylori HopQ interaction.

### hCEACAMs on neutrophils enhance binding, oxidative burst, and phagocytosis of *H. pylori* and modulate bacterial survival in the phagosome.

**FIG 6:**
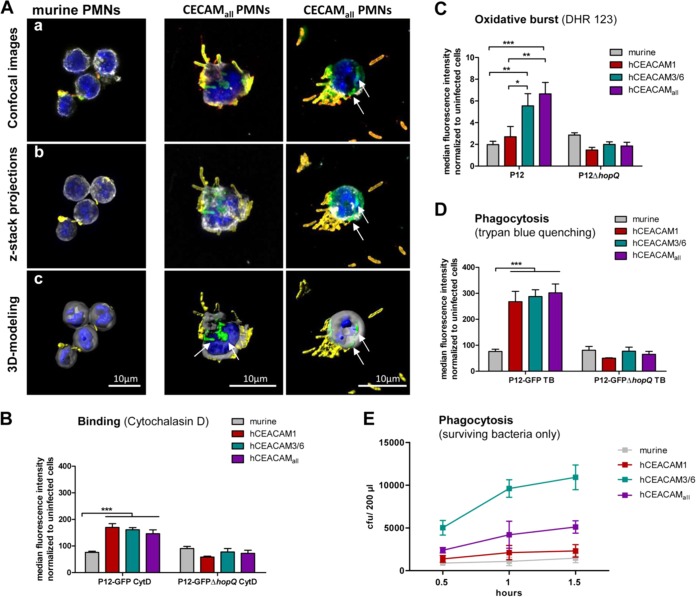
Human CEACAMs on mouse neutrophils promote H. pylori binding, oxidative burst generation, and bacterial internalization and support H. pylori intracellular survival. (Aa) Confocal laser scanning images showing murine PMNs (left) and hCEACAM_all_ PMNs (right) infected with H. pylori after an extracellular/intracellular staining procedure, showing extracellular H. pylori (yellow/orange) and intracellular H. pylori (green). The PMN nucleus is stained by DAPI (blue), and the borders of the cell are labeled with anti-WGA-Alexa647 (gray) (see Materials and Methods for further details). (Ab) Confocal images taken with z-stacks and the projections reconstructed in a three-dimensional cell volume. (Ac) Imaging projections modeled using the Imaris software contour surface tool (see also Video S1). (B) CEACAM-humanized but not wt mouse neutrophils efficiently bind H. pylori in a HopQ-dependent manner, as determined by flow cytometry of cells treated with the actin polymerization inhibitor cytochalasin D to prevent phagocytosis (MOI of 25; 1 h). (C) Mouse wt or CEACAM-humanized neutrophils were infected with H. pylori P12, and oxidative burst responses were analyzed using the fluorescent probe dihydrorhodamine 123 (DHR 123) as described in Materials and Methods. (D) To quantify phagocytosed, intracellular H. pylori, neutrophils were infected with H. pylori P12-GFP or P12Δ*hopQ*-GFP (MOI of 25; 1 h), and the fluorescence of surface-bound extracellular bacteria was quenched with trypan blue. (E) Bacterial survival in neutrophils was evaluated as CFU present in PMN lysates after 1 h of infection after killing of extracellular bacteria and cell perforation (*n* = 3). Data were assessed using two-way ANOVA and the Bonferroni *post hoc* test. *, *P* < 0.05; **, *P* < 0.01; ***, *P* < 0.001.

Unlike wt mouse PMNs, hCEACAM-producing neutrophils efficiently bound H. pylori (Fig. 2A). Confocal laser scanning microscopy of murine PMNs and hCEACAM_all_ PMNs infected with H. pylori and stained for extracellular versus intracellular bacteria pointed to a strong intracellular signal for H. pylori (green) in hCEACAM_all_ PMNs, whereas murine PMNs harbored mainly extracellular or cell-adherent H. pylori (yellow/orange) (Fig. 6A, Video S1). To quantify these events, we applied a flow cytometry analysis. Since bacterial binding and phagocytosis cannot be distinguished by the adhesion assay used, wt and hCEACAM_all_ PMNs were pretreated with cytochalasin D to inhibit phagocytosis prior to infection. H. pylori P12-GFP showed a significantly increased binding to hCEACAM1, hCEACAM3/6, and hCEACAM_all_ expressing PMNs, whereas the P12Δ*hopQ*-GFP strain adhered similarly to wt and CEACAM-humanized neutrophils (Fig. 6B). Cytochalasin D treatment strongly reduced binding of the wt but not the Δ*hopQ* strain (Fig. S7).

10.1128/mBio.03256-19.6 FIG S6 Confocal laser scanning microscopy (CLSM) of hCEACAM_all_ PMNs (A) and murine PMNs (B) showing the individual stainings of the merged image shown in Fig. 2A. (1) hCEACAM_all_ staining with pan CEACAM AB D14HD11, (2) H. pylori-Alexa 488, (3) DAPI, (4) brightfield, (5) merged, (6) merged without brightfield. Download FIG S6, PDF file, 0.7 MB. Copyright © 2020 Behrens et al. 2020 Behrens et al. This content is distributed under the terms of the Creative Commons Attribution 4.0 International license.

10.1128/mBio.03256-19.7 FIG S7 Flow cytometry control experiments for Fig. 6B. (A) Flow cytometry of cells treated with the actin polymerization inhibitor cytochalasin D (CytD) to prevent phagocytosis (MOI of 25; 1 h) compared to cells not treated with CytD (see Fig. 6B). Download FIG S7, PDF file, 0.2 MB. Copyright © 2020 Behrens et al. 2020 Behrens et al. This content is distributed under the terms of the Creative Commons Attribution 4.0 International license.

10.1128/mBio.03256-19.10 VIDEO S1 Intracellular H. pylori after infection of hCEACAM_all_ PMNs. CEACAM_all_ PMNs, as shown in Fig. 6A, are shown as movie to demonstrate the localization of H. pylori after infection. Download Video S1, AVI file, 1.2 MB. Copyright © 2020 Behrens et al. 2020 Behrens et al. This content is distributed under the terms of the Creative Commons Attribution 4.0 International license.

Next, we measured the stimulation of the neutrophil NADPH oxidase and the ability of neutrophils to generate an oxidative burst using the fluorescent dye dihydrorhodamine 123 (DHR 123). Especially hCEACAM_all_ producing PMNs, but also hCEACAM3/6 transgenic neutrophils, but not wt or hCEACAM1 transgenic PMNs revealed a significant oxidative burst, consistent with the function of HopQ protein binding to human CEACAMs (Fig. 6C).

Phagocytosis of H. pylori by human neutrophils and the role of bacterial adhesins has been described before (38, 39), but we were interested to determine the role of hCEACAM receptors in this process. Therefore, we infected isolated wt and hCEACAM transgenic mouse neutrophils with H. pylori P12-GFP and P12Δ*hopQ*-GFP strain and performed phagocytosis experiments. In order to get rid of green fluorescent protein (GFP) fluorescence from adherent bacteria, surface GFP fluorescence was quenched using trypan blue. Consistent with binding, also phagocytosis was significantly enhanced by all three transgenic neutrophil lines compared to wt mouse neutrophils in a HopQ-dependent way (Fig. 6D).

Because hCEACAM transgenic neutrophils can specifically bind and efficiently phagocytose H. pylori (Fig. 6A, B, and D), we wondered whether a functional CEACAM interaction might have an effect for H. pylori survival within activated neutrophils. We therefore applied a plating assay to determine the number of surviving bacteria after bacterial phagocytosis in a time course of infection between 0.5 and 1.5 h. Notably, in neutrophils from CEABAC mice (hCAEACAM3/6) roughly five times more H. pylori survived compared to wt or hCEACAM1-producing neutrophils (Fig. 6E). It appears that hCEACAM1 has an opposite effect on H. pylori survival compared to CEACAM3/6, since neutrophils from hCEACAM_all_ mice showed an intermediate survival rate for H. pylori.

Taken together, our results show that bacterial binding, oxidative burst, and phagocytosis of H. pylori are significantly affected by the interaction of H. pylori HopQ with CEACAMs on PMNs and that H. pylori apparently can modulate its intracellular survival rate by exploiting certain CEACAM receptors.

### The *H. pylori*-CEACAM interaction modulates PMNs to reduce the *H. pylori* CagA translocation efficiency *in vivo*.

Our *in vitro* infection experiments uncovered the important role of a functional H. pylori-CEACAM interaction for the ability of H. pylori to translocate and activate its CagA protein into different myeloid cell types. Next, we were interested in whether the interaction of H. pylori with human CEACAM receptors, as determined with isolated cells *in vitro*, might also have a relevance in the H. pylori mouse model *in vivo* under experimental infection conditions. We therefore infected wt and CEACAM-humanized C57BL/6 mice (hCEACAM_all_ mice) with the mouse-adapted H. pylori strain PMSS1 for 4 weeks and isolated PMNs from the bone marrow of infected and noninfected (naive) animals.

**FIG 7:**
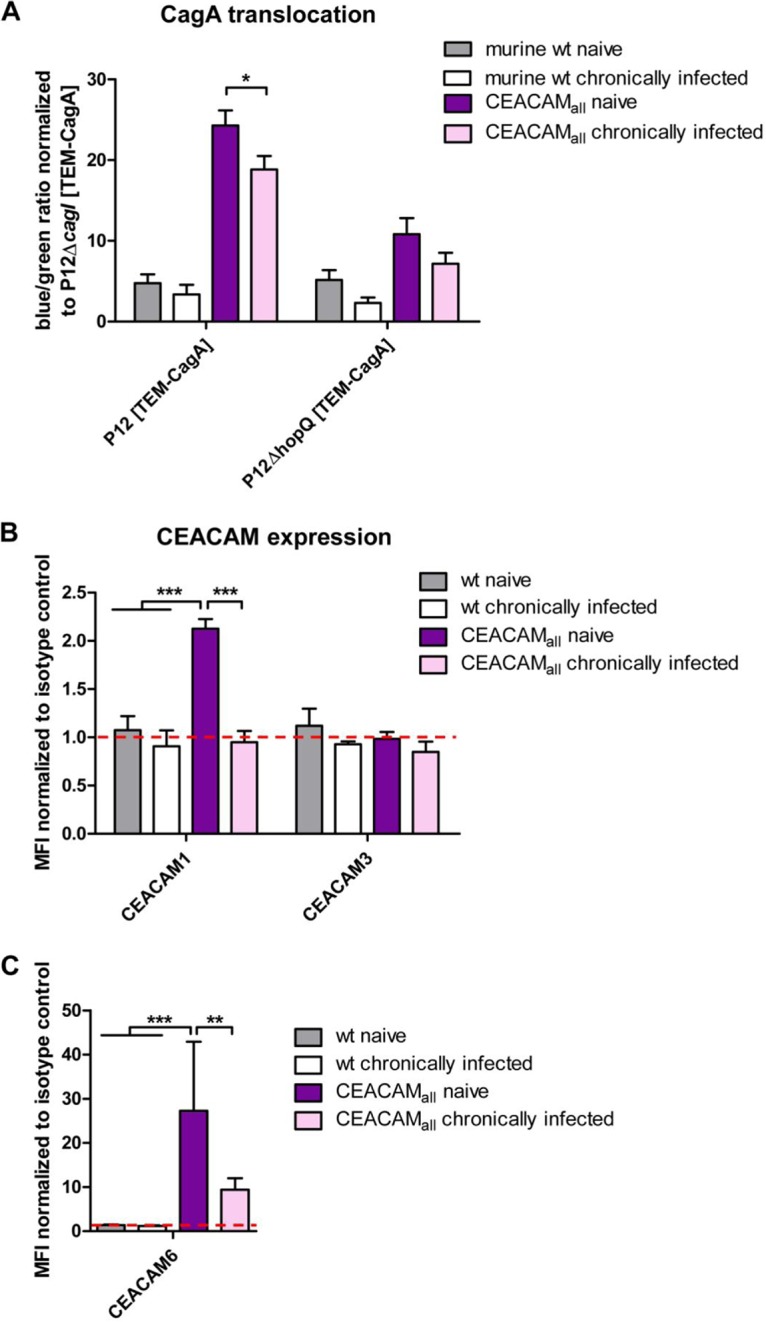
The H. pylori-CEACAM interaction downregulates hCEACAM expression on neutrophils and reduces the H. pylori CagA translocation load after mouse infection *in vivo.* (A) Quantitative evaluation of CagA translocation into neutrophils isolated from naive wt or hCEACAM_all_ mice or chronically infected wt or hCEACAM_all_ mice by the TEM assay. Neutrophils were infected with H. pylori P12[TEM-CagA] or the P12Δ*hopQ*[TEM-CagA] strain (MOI of 60; 2.5 h) and normalized against the translocation-deficient P12Δ*cagI*[TEM-CagA] deletion mutant. (B) Determination of hCEACAM1, hCEACAM3, or hCEACAM6. (C) Production by mouse PMNs isolated from naive wt or hCEACAM_all_ mice or chronically infected wt or hCEACAM_all_ mice was determined by flow cytometry. Mean fluorescence intensity (MFI) was normalized to the isotype control. Data were assessed using two-way ANOVA and the Bonferroni *post hoc* test. *, *P* < 0.05; **, *P* < 0.01; ***, *P* < 0.001.

CagA translocation into neutrophils obtained from age-matched wt naive versus chronically infected animals determined by the TEM assay revealed a low translocation rate, but no significant difference was apparent (Fig. 7A). In contrast, PMNs obtained from CEACAM-humanized animals (hCEACAM_all_) allowed a significantly higher CagA translocation efficiency, consistent with earlier results, but neutrophils obtained from chronically infected hCEACAM_all_ animals showed a significantly reduced CagA translocation compared to PMNs from naive animals (Fig. 7A).

We wondered what might be the physiological basis for this difference and analyzed the hCEACAM surface expression of PMNs isolated from naive and from chronically infected animals by flow cytometry using human CEACAM-specific antibodies. Interestingly, by comparing PMNs from chronically infected animals versus naive CEACAM-humanized mice, we observed a complete loss (hCEACAM1) or a drastic reduction (hCEACAM6) in surface localization of human CEACAMs, respectively (Fig. 7B and C), which could explain the significantly reduced CagA translocation efficiency.

## DISCUSSION

The infection of the human stomach by H. pylori is characterized by a chronic neutrophil-dominant inflammatory response. Mice can be infected with mouse-adapted H. pylori strains only and in contrast to the human H. pylori infection, the H. pylori-infected mouse stomach reveals a much lower immune cell infiltration, resulting in a lower gastric pathology. In humans and in infected mice a H. pylori infection is chronic, and the PMN density correlates with disease severity and tissue destruction in human infection, but the infiltrating PMNs seem to be inefficient toward H. pylori *in vivo* or at least not able to clear the infection (14). Interestingly, H. pylori developed mechanisms to recruit and activate PMNs (39), such as the bacterial protein HP-NAP (40) or the bacterial adhesin SabA, respectively (38). The role of a productive HopQ-CEACAM interaction for the ability of H. pylori to manipulate PMNs, macrophages, or DCs using its major pathogenicity factor, the *cag*-T4SS, and the injection of the effector protein CagA in this respect is not known.

We show here that H. pylori specifically and efficiently interacts with isolated murine PMNs (Fig. 2A) and is able to inject its multifunctional effector protein CagA (Fig. 3). We also demonstrate that injected CagA is not activated by tyrosine phosphorylation, which might have major consequences for the ability of H. pylori to actively manipulate PMNs via CagA phosphorylation-dependent interference with cellular signaling cascades (Fig. 3) (41). About ten host cell proteins, which all harbor so-called Src homology 2 (SH2) domains, are known to bind CagA in a phosphorylation-dependent manner. These include tyrosine phosphatases Shp1 and Shp2, the RasGTPase activating protein (Ras-GAP), phosphoinositide 3-kinase, and the adaptor proteins Crk, Grb2, and Grb7, as well as the tyrosine kinases Csk, c-Src, and c-Abl (42). Thus, as our results suggest, in mouse neutrophils CagA is apparently unable to interfere with SH2 domain-containing cellular proteins and therefore not able to interfere with the same signaling cascades as in human neutrophils. However, CEACAM-humanized mouse PMNs showed (i) a higher translocation of CagA and (ii) a stronger activation by tyrosine phosphorylation compared to wt mouse PMNs (Fig. 3). Furthermore, CagA is processed to an ∼40-kDa phosphorylated C-terminal domain, similar to what occurs in human PMNs (34). The physiological meaning of this processing is thus far unknown.

Furthermore, we provide data that human and mouse wt and CEACAM-humanized DCs and macrophages are able to bind H. pylori, but this interaction seems to occur largely independent of human CEACAMs (Fig. 2C and D). This also reflects the observation that human or CEACAM-humanized DCs or macrophages do either express no (DCs) or only low levels (macrophages) of human CEACAMs on their cell surfaces (Fig. 1A to C). This is in contrast to the expression of endogenous murine CEACAM1 by wt mouse DCs, which express both long and short cytoplasmic variants of CEACAM1 but no other CEACAMs (24).

For both human and murine and CEACAM-humanized DCs we show a low level of CagA translocation and phosphorylation (Fig. 4A and C), whereas the corresponding macrophages revealed a higher CagA phosphorylation level. Both CagA translocation and phosphorylation was independent of the HopQ-CEACAM interaction in these myeloid cells. It is not clear whether these host cells make use of other, as-yet-unknown receptors supporting CagA translocation. This question will be studied in detail in an independent research project in the future.

We also observed that the H. pylori*-*CEACAM interaction controls the production and secretion of chemokines differently in neutrophils, DCs, and macrophages. Using the well-established mouse proinflammatory chemokine panel (BioLegend), we analyzed a set of 13 different chemokines secreted into the supernatant by PMNs, DCs, and macrophages. In particular, chemokines affecting the migration of immune cells to the gastric mucosa were the focus of our research. We therefore concentrated on MIP-1α, KC, and MCP-1 for further analysis. In mouse PMNs or CEACAM-humanized murine PMNs most chemokines tested were at the detection limit or not detected at all. However, MIP-1α was strongly upregulated in CEACAM-humanized PMNs compared to mouse wt PMNs, but not in CEACAM-humanized versus murine DCs or macrophages (Fig. 5A and B). This might be explained by the rather low expression of hCEACAM1 and hCEACAM6 in the latter cells (see Fig. 6 compared to CEACAM-humanized PMNs [Fig. 1A and C]). MIP-1β and LIX are slightly expressed in PMNs, without a significant difference between mouse or CEACAM-humanized PMNs. For DCs, we did not see any significant differences in chemokine expression levels between murine wt and CEACAM-humanized myeloid cells. In macrophages all chemokines analyzed were more or less upregulated upon H. pylori infection, but a strong upregulation was seen for KC, MCP-1, interferon gamma-induced protein 10 (IP10), and to a lower degree macrophage-derived chemokine (MDC) (Fig. S5). In contrast to the situation of MIP-1α in PMNs, KC and MCP-1 were significantly downregulated in CEACAM-humanized versus murine macrophages. All other chemokines analyzed did not differ significantly. This indicates that the interaction of H. pylori with hCEACAM receptors can both upregulate and repress chemokine secretion in different myeloid cell types.

While the H. pylori HopQ-CEACAM interaction is important for bacterial attachment and CagA translocation into different cell types, we analyzed whether hCEACAMs also play a role in the survival of H. pylori in neutrophils. Our data show that stimulation of hCEACAM3/6, rather than the hCEACAM1 receptor on PMNs, support the intracellular survival of H. pylori in neutrophils. Although the oxidative burst by neutrophils interacting with H. pylori was stronger in hCEACAM3/6- and hCEACAM_all_-producing PMNs, is not clear whether the oxygen radicals generated, or non-oxidative components, such as neutrophil proteases or the bactericidal/permeability-increasing protein, might be responsible for this effect (43). However, Neisseria gonorrhoeae is rapidly phagocytosed and inactivated when binding human CEACAM3 on granulocytes, which is accompanied by an intensive oxidative burst generated by CEACAM3-mediated ITAM tyrosine phosphorylation (25, 26). Thus, H. pylori seems to activate a different program in CEACAM-humanized PMNs, allowing its phagocytosis, but to modulate the PMNs to support intracellular survival by as-yet-unknown mechanisms.

The repeated interaction of H. pylori with gastric epithelial cells in pre- and coinfection experiments *in vitro* results in a strong reduction in the CagA translocation efficiency of the later infecting strain (44). We wondered whether neutrophils do respond to an ongoing H. pylori infection in the stomachs of mice and whether a functional HopQ-CEACAM interaction might play a role in the behavior of neutrophils under chronic infection conditions. We therefore isolated PMNs from wt and CEACAM-humanized mice chronically infected by H. pylori PMSS1 (chronically infected PMNs) or not infected (naive PMNs). Interestingly, CEACAM-humanized PMNs from chronically infected animals revealed a significantly reduced CagA translocation rate compared to the corresponding naive PMNs. In contrast, CagA translocation efficiency did not significantly change between naive and chronically infected PMNs obtained from wt mice (Fig. 7A). A possible reason for this neutrophil behavior was a complete abrogation of hCEACAM1 and a strong reduction of CEACAM6 expression on PMNs from chronically infected mice compared to PMNs from naive mice (Fig. 7B and C). Thus, it appears that PMNs can sense the presence of a chronic H. pylori infection via the HopQ-CEACAM interaction or the induced cytokine/chemokine induction and respond by downregulation of CEACAM receptors, eventually reducing the burden of CagA translocation. It will be interesting to analyze whether the same mechanism operates as well in humans.

Generally, a chronic H. pylori infection in humans is negatively associated with the presence of severe asthma in children (45, 46). Furthermore, experimental H. pylori infection of mice, even the administration of a H. pylori cell lysate or its major toxin VacA, protects mice from lung inflammation and allergic airway disease (47). Notably, severe asthma is associated with a strong upregulation of CEACAM6 in human bronchial epithelial cells and especially in neutrophils in the bronchial mucosa, resulting in neutrophil activation, which may contribute to the pathology of the airway disease (48). CEACAM6 expressed by neutrophils may form a homophilic interaction with CEACAM6 overexpressed on airway epithelial cells, which might potentially contribute to neutrophil activation and epithelial damage and dysfunction. Whether the ability of H. pylori to downregulate CEACAM expression on neutrophils isolated and matured from bone marrow could contribute to the beneficial effects of H. pylori on severe asthma in mice and humans has to be tested in future.

## MATERIALS AND METHODS

### Bacterial strains.

H. pylori strains (see Table S2 in the supplemental material) were grown on GC agar plates (Oxoid) supplemented with vitamin mix (1%) and horse serum (8%) (serum plates) and cultured for 16 to 60 h in a microaerobic atmosphere (85% N_2_, 10% CO_2_, 5% O_2_) at 37°C. For TEM assays, H. pylori wild-type strain P12 and defined P12 knockout mutants were used.

10.1128/mBio.03256-19.9 TABLE S2 Bacterial strains used in this study. Download Table S2, DOCX file, 0.02 MB. Copyright © 2020 Behrens et al. 2020 Behrens et al. This content is distributed under the terms of the Creative Commons Attribution 4.0 International license.

### Cultivation of cells.

Cell lines were cultured under standard conditions (50) in 75-cm^2^ tissue culture flasks (BD Falcon) and subcultured every 2 to 3 days. WEHI3B were cultured in RPMI medium with 10% fetal calf serum (FCS).

### Isolation and generation of human neutrophils.

Human PMNs were isolated by density gradient centrifugation from healthy volunteers in accordance with the guidelines from the Declaration of Helsinki and the Ethics Committee of LMU München according to an established protocol (51). In detail, serum (40 ml of heparinized blood) was applied to a Percoll gradient consisting of 4 ml of 74% and 3 ml of 55% Percoll (Sigma). After centrifugation for 20 min at 600 × *g* at room temperature without using a centrifuge brake, the interphase contains the neutrophils.

### Isolation and generation of CEACAM-humanized and wild-type mouse neutrophils.

Murine PMNs were isolated by density gradient centrifugation, as described previously (52, 53). In brief, the femurs and tibias were dissected and incubated for 5 min in 70% ethanol for disinfection. Afterward, the ends of the bones were cut, and the cells were flushed with PBS with a syringe. A Percoll gradient was prepared consisting of 52, 64, and 72% Percoll. The cells were layered on the top of the gradient and subsequently centrifuged without using a centrifuge brake. Cells between the 64 and 72% interphase were collected, washed once with PBS, and then cultured overnight in RPMI with 20% interleukin-3 (IL-3) containing supernatant of WEHI-3B cells.

### Isolation and generation of CEACAM-humanized and wild-type DCs and macrophages.

Murine DCs and macrophages were isolated and generated according to the protocol of Lutz et al. (54). In brief, bone marrow samples were isolated as described for the isolation of PMNs. A total of 5 × 10^6^ cells were cultured in a petri dish with media containing 20 ng/ml recombinant mouse granulocyte-macrophage colony-stimulating factor (GM-CSF) to generate DCs and 10 ng/ml recombinant mouse M-CSF to generate macrophages for 10 to 12 days after stimulation.

### Isolation of human DCs and macrophages.

Monocytes were isolated from whole blood from healthy volunteers. In detail, 20 ml of human blood was mixed with 30 ml of PBS and layered on a Ficoll gradient. After 30 min of constant centrifugation at 400 × *g* without using a centrifuge brake mononuclear layer was collected. The cells were washed and resuspended in 100 μl of PBS. Then, 20 μl of MACS CD14 beads was added, followed by incubation at 4°C for 15 min. Next, 15 ml of PBS was added, followed by incubation at 4°C for 10 min at 350 × *g*. The cells were resuspended in 500 μl of PBS and transferred to an equilibrated MACS column (Miltenyi Biotec). After a wash with PBS, the cells were eluted and cultured in RPMI medium with 2% FCS, 0.05% recombinant human GM-CSF (ImmunoTools), and 0.02% IL-4 for 6 days to generate DCs. For generation of macrophages 50 ng/ml recombinant human M-CSF (ImmunoTools) were used. New medium was added every other day.

### Determination of CEACAM expression by flow cytometry.

Cells were counted and added to a round-bottom 96-well plate with 2 × 10^5^ cells per well. After centrifugation (300 × *g* at 4°C), primary antibodies were added to each well according to the recommended concentration from the manufacturer. Dilutions of antibody, if necessary, were made in fluorescence-activated cell sorting (FACS) buffer. Cells and antibodies were incubated at 4°C for 1 h in the dark. Primary antibody-stained cells were washed three times and resuspended in 200 μl to 1 ml of ice-cold FACS buffer. Subsequently, fluorochrome-labeled secondary antibodies were diluted in FACS buffer at the optimal concentration (according to the manufacturer’s instructions) and then added to each well, followed by 1 h of incubation at 4°C in the dark and three washes as described above. The cells were analyzed by flow cytometer right after washing or kept in the dark on ice until the scheduled time for analysis. The indicated antibodies were used: hCEACAM1 (4/3/17; Genovac), hCEACAM3 (col-1; Invitrogen), and hCEACAM6 (9A6; Genovac).

### Quantitative evaluation of CagA translocation into human, murine, and CEACAM-humanized PMNs and DCs by a TEM assay.

A total of 2 × 10^5^ cells per well were used. Before infection, H. pylori strains with a β-lactamase TEM-CagA fusion protein were collected as described before. Ideally, bacteria were resuspended and preincubated in sterile PBS containing 10% FCS at 37°C and 10% CO_2_ for 1.5 h. Subsequently, the cells were infected with bacteria with a multiplicity of infection (MOI) of 60 for 2.5 h at 37°C and 5% CO_2_ as described above. Infections were stopped by placing the plates on ice and removing the supernatants. The prepared substrate mixes were loaded into the cells, followed by incubation at room temperature for 120 min in the dark. Finally, the cells were washed at least twice with PBS by centrifugation at 200 × *g* to 300 × *g* for 5 min after incubation with CCF4-AM substrate. Cells were then analyzed by flow cytometry for Pacific Blue fluorescence and AmCyan green fluorescence. The mean fluorescence intensity was normalized to the noninfected cells samples.

### Translocation of CagA determined by tyrosine-phosphorylation of CagA in the immunoblot.

A total of 5 × 10^5^ cells were infected with an MOI of 60. The infection was stopped after 3 h on ice. Cells were collected in PBS containing 1 mM sodium vanadate, 1 μM leupeptin, 1 μM pepstatin, and 1 mM phenylmethylsulfonyl fluoride and resuspended in 2× SDS loading buffer. Equal amounts of samples were loaded on a gel and analyzed by Western blotting.

### Analysis of *in vitro* phosphorylation of CagA in murine cell lysates.

A total of 6 × 10^6^ cells were centrifuged and resuspended in NP-40 (20 mM Tris-HCl [pH 7.5], 150 mM NaCl, 1% Nonidet P-40], 1 mM EDTA [pH 8], 1 mM sodium-vanadate, 1 mM phenylmethylsulfonyl fluoride, 1 μM leupeptin, 1 μM pepstatin). Next, 45 μl of cells, 45 μl of bacteria, and 11 μl of phosphorylation buffer (1 ml of 10× phosphorylation buffer = 250 mM Tris-HCl [pH 7.2], 312.5 mM MgCl_2_ ⋅ H_2_O, 62.5 mM MnCl_2_ ⋅ 4H_2_O, and 1 mM sodium vanadate), and 400 μM ATP were incubated 10 min at 30°C. Then, 28 μl of 5× SDS sample buffer was added. Equal amounts of samples were loaded onto a gel and analyzed by Western blotting.

### Sodium dodecyl sulfate-polyacrylamide gel electrophoresis and Western blotting.

SDS-PAGE and Western blotting was performed as described previously (8). For the development of immunoblots, polyvinylidene difluoride (polyvinylidene difluoride) filters were blocked with 5% nonfat milk powder in TBS (50 mM Tris-HCl [pH 7.5], 150 mM NaCl) and 0.1% (vol/vol) Tween 20 (TBS-T) and then incubated with the respective antisera at a dilution of 1:1,000 to 1:15,000 in TBS-T with 3% bovine serum albumin (BSA). Horseradish peroxidase-conjugated anti-rabbit IgG antiserum was used to visualize the bound antibody. Standard infections of with H. pylori strains and subsequent preparations for phosphotyrosine immunoblotting were performed as described previously (8). CagA protein was detected using α-CagA antibody 299. Tyrosine-phosphorylated proteins were analyzed by immunoblotting with phosphotyrosine antibody PY99 (Santa Cruz Biotechnologies).

### Stain-Free staining.

Single gel systems (55) were adapted for Stain-Free detection as described in protocol depository Protocols.io (https://doi.org/10.17504/protocols.io.gipbudn).

### Analysis of the interaction of *H. pylori* with neutrophils, DCs, and macrophages.

H. pylori strains were stained with DAPI (4′,6′-diamidino-2-phenylindole; 5 μg/ml) for 10 min. Afterward, the cells were infected with DAPI-stained H. pylori or GFP-expressing H. pylori at an MOI of 10 for 1 h at 37°C and 5% CO_2_. After three washes with PBS containing 2 mM EDTA, 0.5% FCS, and 0.1% BSA, the cells were collected and analyzed in a flow cytometer (FACS CantoII; BD Biosciences). The median fluorescence intensities were normalized to uninfected cells and GFP or DAPI expression.

### Binding of *H. pylori* to neutrophils.

Binding was studied in neutrophils, which were incubated with 10 μg/ml cytochalasin D to inhibit phagocytosis. These neutrophils were infected with H. pylori expressing GFP for 1 h at an MOI of 25. The cells were then washed, and the binding was analyzed by flow cytometry.

### Phagocytosis of *H. pylori* by neutrophils.

Phagocytosis was analyzed by flow cytometry by quenching the signal of extracellular adherent bacteria, as described previously (56). In detail, cells were infected with H. pylori at an MOI of 25 for 1 h. Afterward, the cells were washed with flow cytometry buffer. Directly before the analysis by flow cytometry, trypan blue was added. In this way the signal of the extracellular bacteria was quenched, and only the fluorescence of intracellular bacteria was detected.

### Analysis of bacteria surviving the phagocytosis.

The gentamicin killing assay is a method based on that of Odenbreit et al. (9) to analyze the survival of phagocytosed bacteria. In detail, cells were infected with H. pylori at an MOI of 25 for 1 h. The extracellular bacteria were then killed by gentamicin by 1 h incubation, followed by washing and permeabilization of the cells with saponin. Afterward, the supernatant was plated on serum plates and, after a few days, the CFU were counted.

### Measurement of the production of reactive oxygen species of *H. pylori*-infected neutrophils.

According to the method of Chen et al., the production of reactive oxygen species was measured using dihydrorhodamine 123 by flow cytometry (57). Dihydrorhodamine 123 is a nonfluorescent dye that is oxidized by reactive oxygen production to green fluorescent rhodamine. Therefore, PMNs were infected with H. pylori at an MOI of 60. Then, 2.8 μl of a 30 mM stock dihydrorhodamine 123 was added to the infection. After incubation at 37°C for 20 min, the infected cells were centrifuged and fixed with PBS containing 2 mM EDTA, 0.5% FCS, 0.1 BSA, and 4% paraformaldehyde (PFA). Finally, the green fluorescence of rhodamine was analyzed in a flow cytometer (FACS CantoII).

### Microscopy of *H. pylori* binding to neutrophils.

PMNs were infected with H. pylori wt or isogenic mutant strains at an MOI of 10 for 1h at 37°C and 5% CO_2_. After infection, 2 × 10^5^ PMNs were transferred to a slide by using a cytospin (cytospin3; Shandon) on a microscope slide. For immunostaining cells were fixed with 4% PFA for 10 min at room temperature. The cells were washed twice with Dulbecco PBS (Life Technologies) and blocked overnight with 2% FCS in PBS at 4°C. Fixed cells were incubated with rabbit anti-H. pylori (AK175; 1:400), mouse anti-CEACAM (panCEACAM; Genovac; 1:200), and anti-agglutinin (WGA)-Alexa 647 for 1 h at room temperature. After a washing step, secondary antibodies were applied (goat anti-rabbit Alexa 488 from Invitrogen [1:1,000]) and goat anti-mouse Alexa 555, followed by incubation for 1 h at room temperature in the dark. Cell nuclei were stained with DAPI (5 μg/ml) for 10 min. Samples were mounted on the coverslip with fluorescent mounting medium (Dako). Micrographs were taken with a confocal laser scanning microscope (LSM880; Zeiss) with Airyscan module using a 63× oil immersion objective.

### Microscopy of *H. pylori* binding on and phagocytosed in neutrophils (extracellular/intracellular staining procedure).

Microscopy was performed as described above. Infected cells were incubated with anti-WGA-Alexa 647 for neutrophil labeling and rabbit anti-H. pylori antibody (AK175, 1:400); then, after a washing, the secondary antibody (goat anti-rabbit Alexa 555 from Invitrogen [1:1,000]) was applied, followed by incubation for 1 h at room temperature in the dark. The cells were then permeabilized with Triton X-100 to visualize internalized H. pylori in the cell and then incubated again with rabbit anti-H. pylori (AK175; 1:400). After washing, secondary antibody was applied (goat anti-rabbit Alexa 488 from Invitrogen [1:1,000]), followed by incubation for 1 h at room temperature in the dark. Thus, extracellular bacteria are labeled red and green, resulting in yellow/orange, whereas intracellular bacteria are green. Confocal images were taken with z-stack, and the projections were reconstructed in a three-dimensional cell volume by using Imaris software. The imaging projections were rendered using the Imaris software contour surface tool.

### Determination of chemokine secretion by using the LEGENDplex mouse proinflammatory chemokine 13-plex panel.

A total of 5 × 10^5^ cells were infected with H. pylori at an MOI of 60. After 3 h, infection supernatants were collected. Chemokine concentrations were measured by using mouse proinflammatory chemokine 13-plex panel (BioLegend) according to the instructions of the manufacturer. Data were analyzed with LEGENDplex software.

### Mouse infections.

Specific-pathogen-free mice (Mus musculus) were bred, infected, and sacrificed at the Max von Pettenkofer Institute (Munich, Germany). CEACAM-humanized mice were established as previously described (32, 33, 58) and combined by breeding. C57BL/6J mice served as controls. All experiments and procedures were conducted in accordance with the *Guidelines for the Care and Use of Laboratory Animals* and approved by the Regierung von Oberbayern (ROB-55.2-2532.Vet_02-18-189). The animals (males and females) were infected orogastrically two times on subsequent days at a dose of 10^9^ bacteria. Samples were obtained from animals as follows. The animals were killed 4 weeks after infection by using CO_2_. Stomachs were opened along the greater curvature and washed with PBS. The tissue was homogenized using a glass homogenizer (Ochs) in brucella broth medium (Difco) to isolate bacteria. Homogenates were spread in appropriate dilutions on selective agar plates (GC agar [Difco] including 10% horse serum [Gibco] and DENT supplementation [Oxoid]). The number of CFU was calculated per gram of gastric tissue.

### Statistics.

Data were obtained from at least three independent experiments. Statistical analyses were performed with GraphPad Prism 5. Significance between independent groups was calculated using two-way analysis of variance (ANOVA) and the Bonferroni *post hoc* test.
