# Supplementary material for: The HopQ-CEACAM Interaction Controls CagA Translocation, Phosphorylation, and Phagocytosis of Helicobacter pylori in Neutrophils
Source: mBio. 2020 Feb 4;11(1):e03256-19. doi: 10.1128/mBio.03256-19 (PMC7002351; doi:10.1128/mBio.03256-19)
Supplement: TABLE S1 [file mBio.03256-19-st001.docx]

**Table S1: Antibodies used in this study and their source**

| **Antibody name** | **Company or Reference** | |
| --- | --- | --- |
| CEACAM1 (4/3/17) | Genovac | |
| CEACAM3 (col-1) | Invitrogen | |
| CEACAM6 (9A6) | Genovac | |
| D14HD11 pan CEACAM | Genovac | |
| APC rat α mouse Ly6G (1A8) | Biolegend | |
| FITC α mouse/human CD11b (M1/70) | Biolegend | |
| PE α mouse CD11c (HL3) | BD BioScience | |
| PE Cy5 α mouse F4/80 (Bm8) | e Bio Science | |
| Rat IgG2a κ isotype control APC (eBr2a) | Biolegend | |
| Rat IgG2a κ isotype control FITC | BD BioScience | |
| Rat IgG2a κ isotype control PE (eBr2a) | e Bio Science | |
| Rat IgG2a κ isotype control PE Cy5 (eBr2a) | e Bio Science | |
|  |  |  |
|  |  |  |
