# Supplementary figures and images for: The HopQ-CEACAM Interaction Controls CagA Translocation, Phosphorylation, and Phagocytosis of Helicobacter pylori in Neutrophils
Source: mBio. 2020 Feb 4;11(1):e03256-19. doi: 10.1128/mBio.03256-19 (PMC7002351; doi:10.1128/mBio.03256-19)

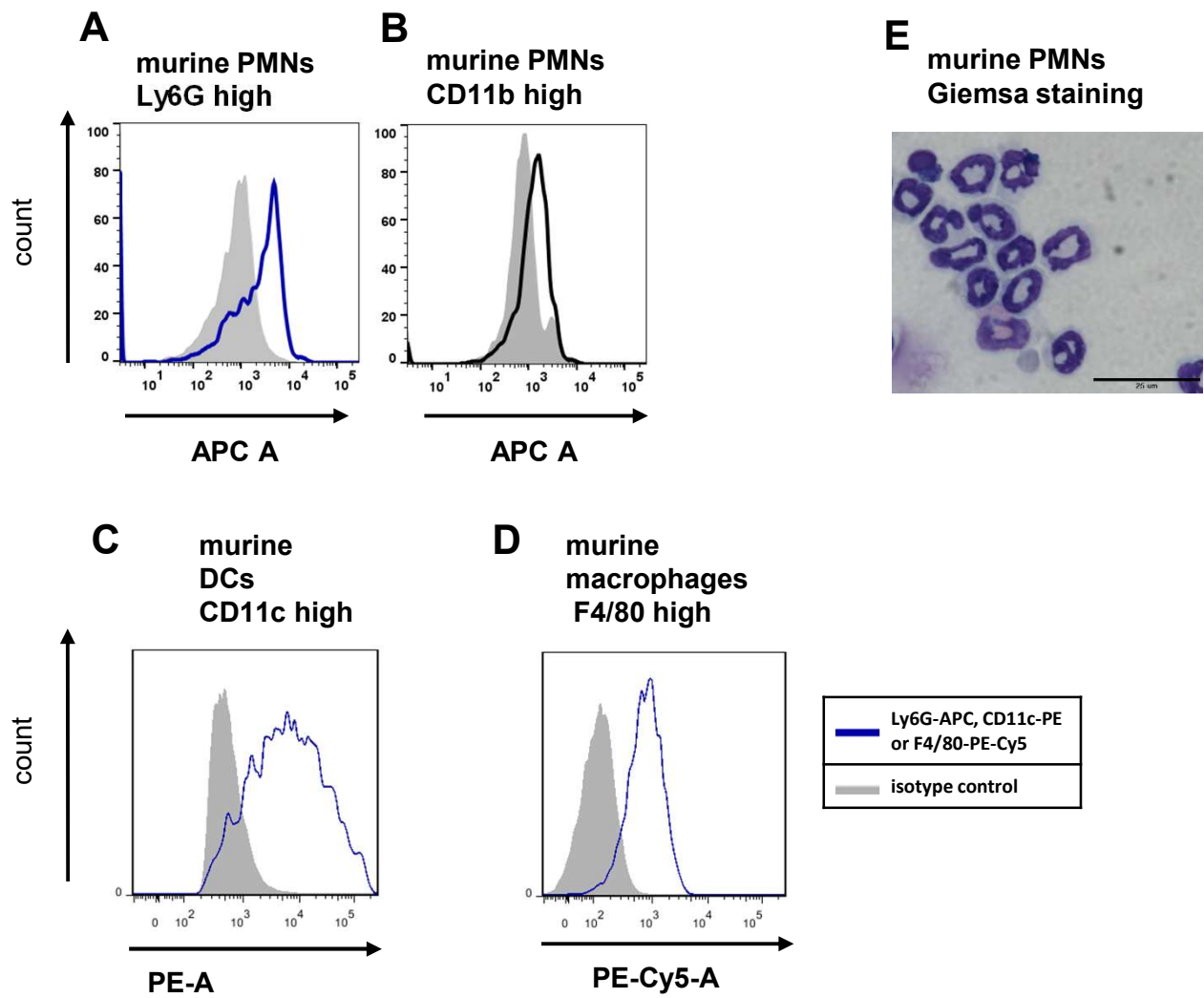

Supplement: FIG S1 [file mBio.03256-19-sf001.pdf]

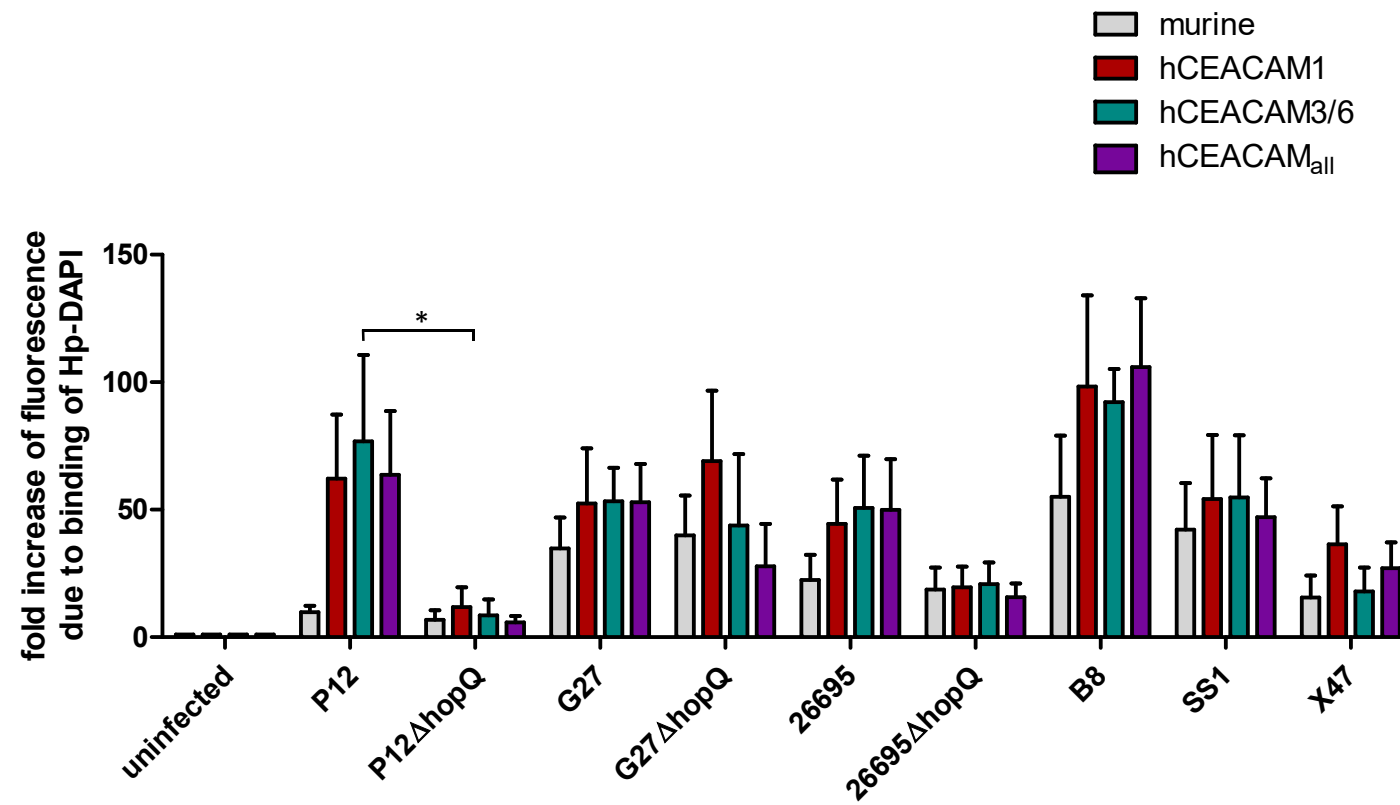

Supplement: FIG S2 [file mBio.03256-19-sf002.pdf]

## *In vitro* CagA phosphorylation assay

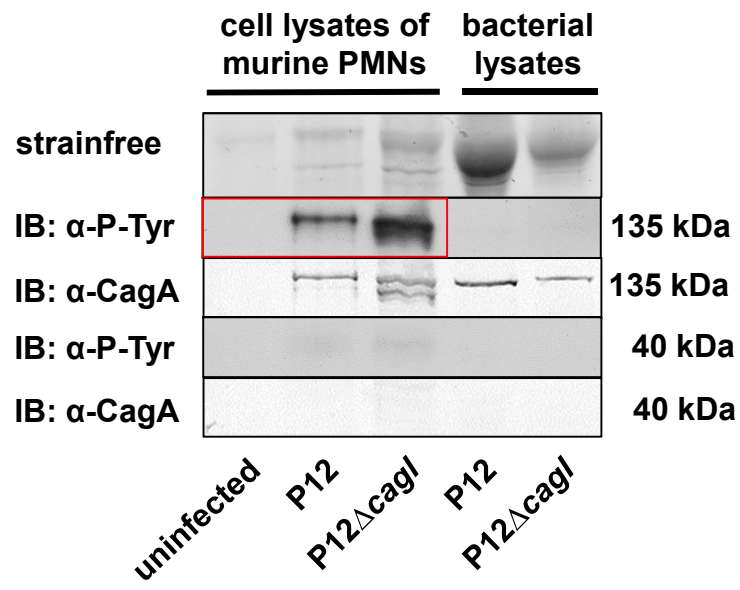

Behrens et al., Fig. S3

Supplement: FIG S3 [file mBio.03256-19-sf003.pdf]

## Comparison TEM-CagA assay for murine DCs and macrophages

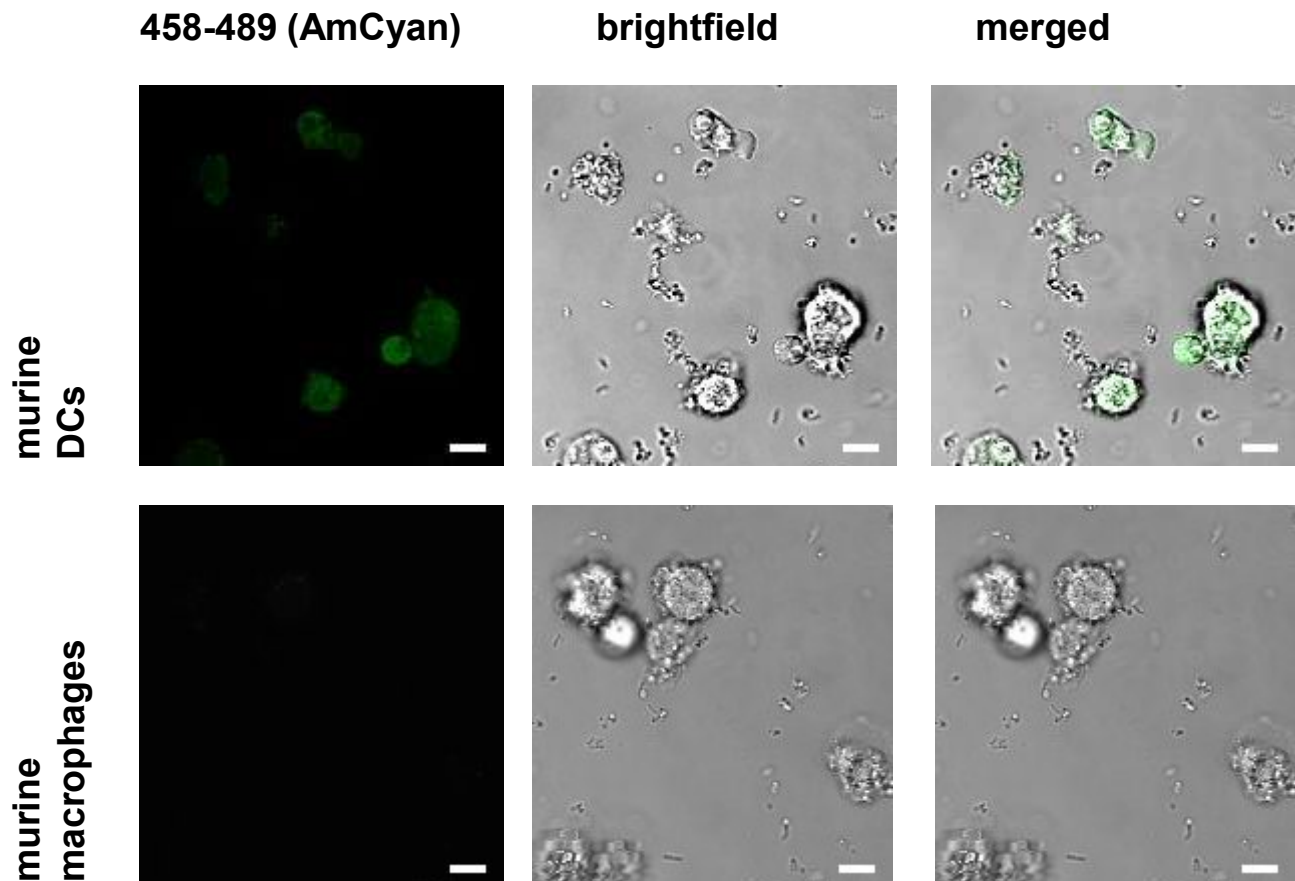

Supplement: FIG S4 [file mBio.03256-19-sf004.pdf]

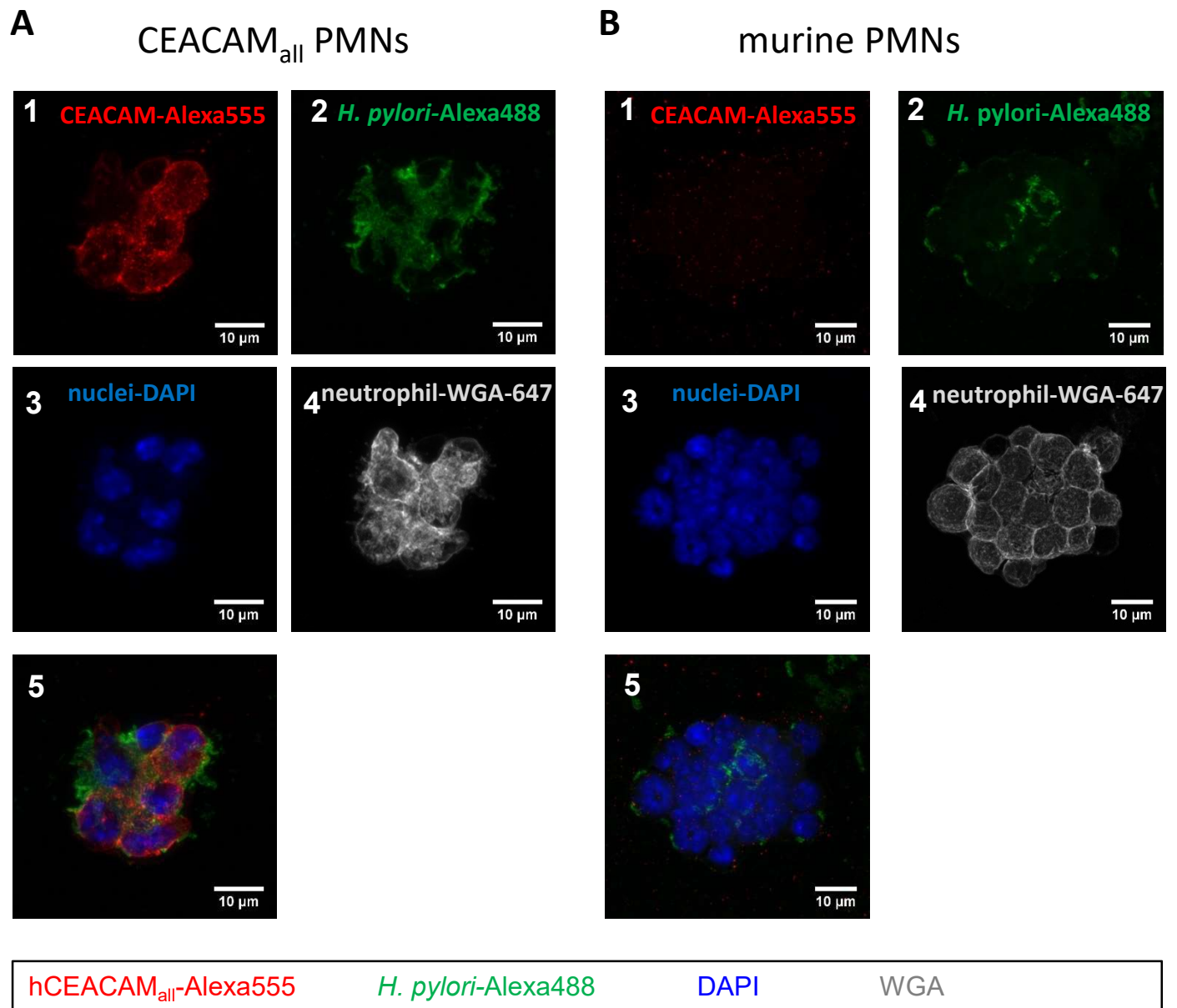

Supplement: FIG S6 [file mBio.03256-19-sf006.pdf]

Binding (cytochalasin D control)

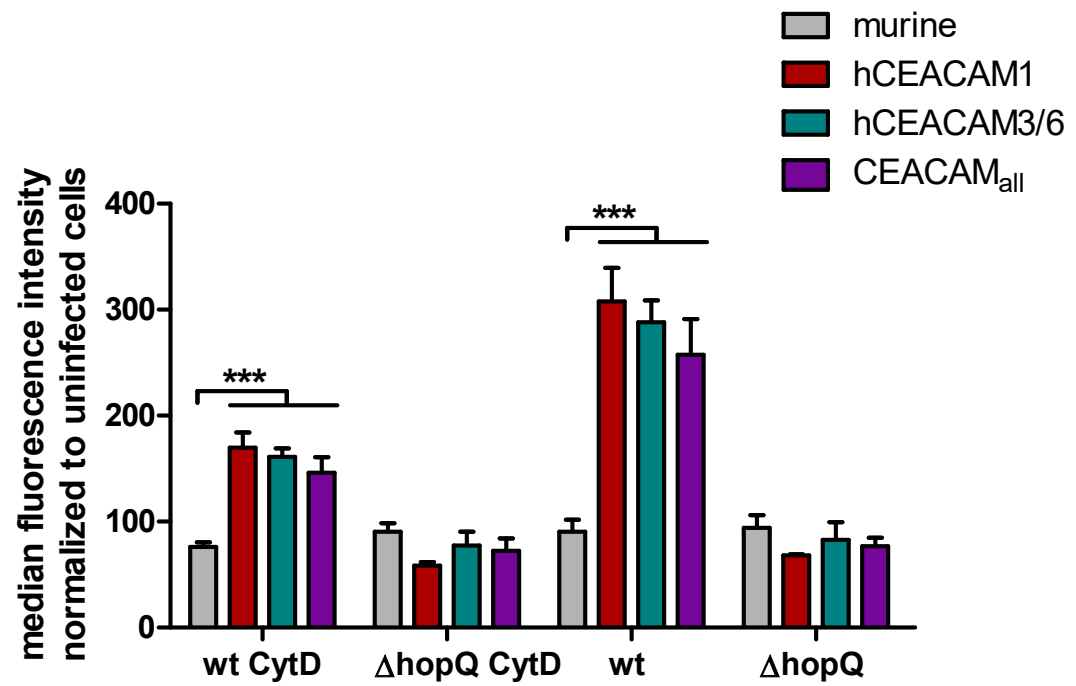

Supplement: FIG S7 [file mBio.03256-19-sf007.pdf]
