# Supplementary material for: The HopQ-CEACAM Interaction Controls CagA Translocation, Phosphorylation, and Phagocytosis of Helicobacter pylori in Neutrophils
Source: mBio. 2020 Feb 4;11(1):e03256-19. doi: 10.1128/mBio.03256-19 (PMC7002351; doi:10.1128/mBio.03256-19)
Supplement: TABLE S2 [file mBio.03256-19-st002.docx]

**Table S2: Bacterial strains used in this study**

| **Strain** | **Special features of strain** | **Reference** |
| --- | --- | --- |
| ***Hp* strains and mutants** |  |  |
| P12 |  | (1) |
| P12Δ*hopQ* | Deletion of complete *hopQ* gene | (2) |
| P12*∆cagI* | Deletion of *cagI* gene, defective T4SS | (3) |
| P12-GFP | Green fluorescent *H. pylori* strain used for PMN binding studies by flow cytometry | (2) |
| P12∆*hopQ* GFP | GFP-labelled *hopQ* mutant strain for PMN binding studies | (2) |
| P12 [TEM-CagA] | TEM-CagA producing P12 strain to measure and quantify CagA translocation | (4) |
| P12*∆hopQ* [TEM-CagA] | Same as P12 [TEM-CagA] with deletion of *hopQ* gene | (5) |
| P12*∆cagI* [TEM-CagA] | Deletion of *cagI* gene, defective T4SS, but production of a TEM-CagA protein | (4) |
| 26695 | wt *H. pylori* strain | (6) |
| 26695*∆hopQ* | wt *H. pylori* strain with deletion of *hopQ* | This study |
| G27 | wt *H. pylori* strain | (7) |
| G27*∆hopQ* | wt *H. pylori* strain with deletion of *hopQ* | This study |
| B8 | wt *H. pylori* strain, able to infect Mongolian gerbils | (8) |
| SS1 | wt *H. pylori* strain, able to infect mice | (9) |
| X47 | wt *H. pylori* strain, able to infect mice | (10) |
|  |  |  |

**References**

1. Schmitt W, Haas R. 1994. Genetic analysis of the *Helicobacter pylori* vacuolating cytotoxin: structural similarities with the IgA protease type of exported protein. Mol Microbiol 12:307-319.

2. Königer V, Holsten L, Harrison U, Busch B, Loell E, Zhao Q, Bonsor DA, Roth A, Kengmo-Tchoupa A, Smith SI, Mueller S, Sundberg EJ, Zimmermann W, Fischer W, Hauck CR, Haas R. 2016. *Helicobacter pylori* exploits human CEACAMs via HopQ for adherence and translocation of CagA. Nat Microbiol 2:16188-16199.

3. Pham KT, Weiss E, Jimenez-Soto LF, Breithaupt U, Haas R, Fischer W. 2012. CagI Is an Essential Component of the *Helicobacter pylori* Cag Type IV Secretion System and Forms a Complex with CagL. PLoS One 7:e35341.

4. Schindele F, Weiss E, Haas R, Fischer W. 2016. Quantitative analysis of CagA type IV secretion by *Helicobacter pylori* reveals substrate recognition and translocation requirements. Mol Microbiol 100:188-203.

5. Zhao Q, Busch B, Jimenez-Soto LF, Ishikawa-Ankerhold H, Massberg S, Terradot L, Fischer W, Haas R. 2018. Integrin but not CEACAM receptors are dispensable for *Helicobacter pylori* CagA translocation. PLoS Pathog 14:e1007359.

6. Tomb J-F, White O, Kerlavage AR, Clayton RA, Sutton GG, Fleischmann RD, Ketchum KA, Klenk HP, Gill S, Dougherty BA, Nelson K, Quakenbush J, Zhou L, Kirkness EF, Peterson S, Loftus B, Richardson D, Dodson R, Khalak HG, Glodek A, McKenney K, Fitzegerald LM, Lee N, Adams MD, Hickey EK, Berg DE, Gocayne JD, Utterback TR, Peterson JD, Kelley JM, Cotton MD, Weidman JM, Fujii C, Bowman C, Watthey L, Wallin E, Hayes WS, Borodovsky M, Karp PD, Smith HO, Fraser CM, Venter JC. 1997. The complete genome sequence of the gastric pathogen *Helicobacter pylori*. Nature 388:539-547.

7. Covacci A, Censini S, Bugnoli M, Petracca R, Burroni D, Macchia G, Massone A, Papini E, Xiang Z, Figura N, Rappuoli R. 1993. Molecular characterization of the 128-kDa immunodominant antigen of *Helicobacter pylori* associated with cytotoxicity and duodenal ulcer. Proc Natl Acad Sci USA 90:5791-5795.

8. Farnbacher M, Jahns T, Willrodt D, Daniel R, Haas R, Goesmann A, Kurtz S, Rieder G. 2010. Sequencing, annotation and comparative genome analysis of the gerbil-adapted *Helicobacter pylori* strain B8. BMC Genomics 11:335.

9. Lee A, O´Rourke J, de Ungria MC, Robertson B, Daskalopoulos G, Dixon MF. 1997. A standardized mouse model of *Helicobacter pylori* infection: introducing the sydney strain. Gastroenterology 112:1386-1397.

10. Kleanthous H, Tibbitts TJ, Gray HL, Myers GA, Lee CK, Ermak TH, Monath TP. 2001. Sterilizing immunity against experimental *Helicobacter pylori* infection is challenge-strain dependent. Vaccine 19:4883-4895.
