# Supplementary material for: The HopQ-CEACAM Interaction Controls CagA Translocation, Phosphorylation, and Phagocytosis of Helicobacter pylori in Neutrophils
Source: mBio. 2020 Feb 4;11(1):e03256-19. doi: 10.1128/mBio.03256-19 (PMC7002351; doi:10.1128/mBio.03256-19)
Supplement: FIG S5 [file mBio.03256-19-sf005.pdf]

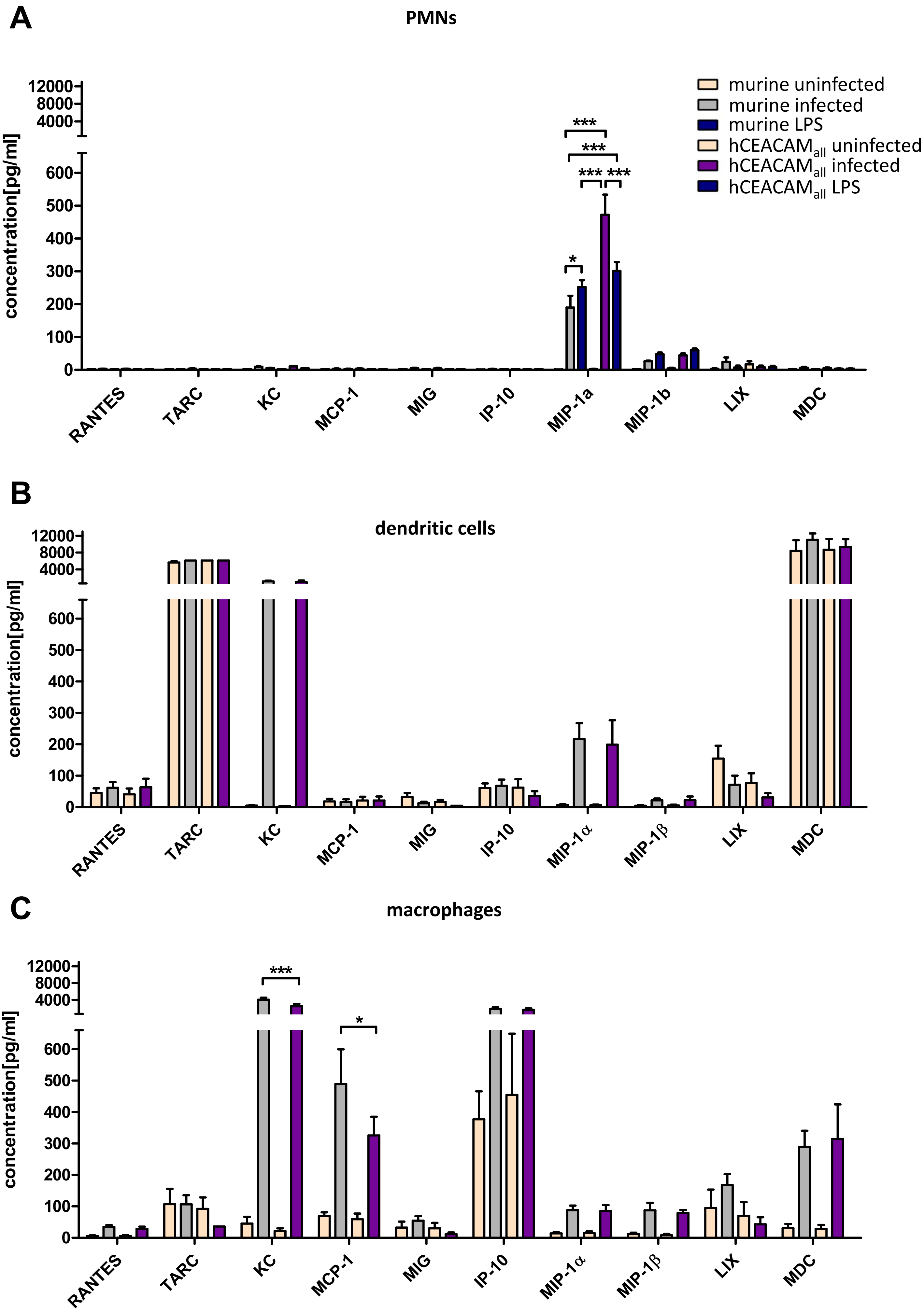

Behrens et al Fig. S 5
